# A SURF4-to-proteoglycan relay mechanism that mediates the sorting and secretion of a tagged variant of sonic hedgehog

**DOI:** 10.1073/pnas.2113991119

**Published:** 2022-03-10

**Authors:** Xiao Tang, Rong Chen, Vince St Dollente Mesias, Tingxuan Wang, Ying Wang, Kristina Poljak, Xinyu Fan, Hanchi Miao, Junjie Hu, Liang Zhang, Jinqing Huang, Shuhuai Yao, Elizabeth A. Miller, Yusong Guo

**Affiliations:** ^a^Division of Life Science and State Key Laboratory of Molecular Neuroscience, The Hong Kong University of Science and Technology, Hong Kong, China;; ^b^Department of Chemical and Biological Engineering, The Hong Kong University of Science and Technology, Hong Kong, China;; ^c^Department of Chemistry, The Hong Kong University of Science and Technology, Clear Water Bay, Hong Kong, China;; ^d^Department of Biomedical Sciences, The City University of Hong Kong, Hong Kong, China;; ^e^Cell Biology Division, Medical Research Council Laboratory of Molecular Biology, Cambridge CB2 0QH, United Kingdom;; ^f^National Laboratory of Biomacromolecules, Chinese Academy of Sciences Center for Excellence in Biomacromolecules, Institute of Biophysics, Chinese Academy of Sciences, Beijing 100101, China;; ^g^Department of Mechanical and Aerospace Engineering, The Hong Kong University of Science and Technology, Hong Kong, China;; ^h^Shenzhen Research Institute, Hong Kong University of Science and Technology, Shenzhen 518057, China;; ^i^Southern Marine Science and Engineering Guangdong Laboratory (Guangzhou), Guangzhou 511458, China

**Keywords:** COPII, cargo receptor, cargo sorting, SURF4, ER

## Abstract

Sonic Hedgehog (Shh) is a key signaling molecule that plays important roles in various developmental processes in mammals. Although the signal transduction pathway activated by Shh is well understood, the regulation of its secretion remains unclear. Newly synthesized Shh is imported into the endoplasmic reticulum (ER), where it undergoes a series of posttranslational modifications to produce the mature lipid-modified amino-terminal fragment. Here, we have analyzed the molecular mechanisms that mediate secretion of the N-terminal fragment of Shh (ShhN). We found that the Cardin–Weintraub (CW) motif in Shh is necessary and sufficient for ER-to-Golgi transport of ShhN. Mechanistic analyses revealed that a cargo receptor, Surfeit locus protein 4 (SURF4), interacts directly with the CW motif of ShhN to regulate packaging of ShhN into COPII vesicles. ShhN and SURF4 interact with each other at the ER and separate from each other after entering the Golgi. The CW motif is known to interact with proteoglycans (PGs) that are predominantly synthesized at the Golgi. Interestingly, we found that PGs compete with SURF4 to bind ShhN and that inhibiting synthesis of PGs causes defects in export of ShhN from the *trans* Golgi network (TGN). SURF4 and PG maturation are also important for intracellular traffic of full length Shh in mammalian cells. Our study suggests a SURF4-to-PG relay mechanism that mediates the sorting and secretion of Shh, providing insight into the biosynthetic trafficking of Shh.

## Significance

Sonic Hedgehog (Shh) is a key signaling molecule that plays important roles in embryonic patterning, cell differentiation, and organ development. Although fundamentally important, the molecular mechanisms that regulate secretion of newly synthesized Shh are still unclear. Our study reveals a role for the cargo receptor, SURF4, in facilitating export of Shh from the endoplasmic reticulum (ER) via a ER export signal. In addition, our study provides evidence suggesting that proteoglycans promote the dissociation of SURF4 from Shh at the Golgi, suggesting a SURF4-to-proteoglycan relay mechanism. These analyses provide insight into an important question in cell biology: how do cargo receptors capture their clients in one compartment, then disengage at their destination?

The Hedgehog (Hh) signaling pathway plays an important role in various developmental processes in metazoans (1, 2). Mutations of key components that regulate Hh signaling are associated with many human diseases (3). Hh was first found in the *Drosophila* larval epidermis. It mediates larval segment development and adult appendage patterning (4). In mammals, there are three Hh-family members, Sonic hedgehog (Shh), Indian hedgehog (Ihh), and Desert hedgehog (Dhh). Ihh regulates the proliferation and differentiation of chondrocytes (5). Dhh functions in gonads, regulating testis organogenesis, spermatogenesis (6, 7), and follicle development in the ovary (8). Shh functions more extensively than the other two Hh members: it regulates embryonic patterning (4), specification of cell types in the nervous system (9), axon guidance (10), cell differentiation, and organ development (11).

Hh is synthesized as a full-length precursor Hh (HhFL). After entering the endoplasmic reticulum (ER), HhFL is autocleaved into two parts: an N-terminal Hedge domain (HhN) and a C-terminal Hog domain (HhC) (1). HhC is degraded through ER-associated degradation (12). HhN undergoes lipid modifications, in which a cholesterol molecule is covalently linked to the C terminus and a palmitoyl group is linked to the N terminus (13–15). Lipid-modified HhN subsequently exits the ER and is delivered via the secretory pathway to the plasma membrane. Once at the plasma membrane, Hh is released into the extracellular matrix and ultimately recognized by its receptors on the plasma membrane of target cells to induce downstream signal transduction.

Although significant progress has been achieved in understanding the Hh signaling pathway in target cells, the molecular mechanisms that mediate secretion of newly synthesized Shh proteins from the producing cells are still unclear. The ER is the first station where newly synthesized proteins enter the secretory pathway. In this compartment, cargo proteins are generally recognized by the coat protein complex II (COPII) to be packaged into vesicles and exported from the ER. Soluble cargo proteins in the ER lumen cannot directly engage the COPII coat but instead are captured into vesicles by transmembrane cargo receptors. One mammalian cargo receptor, ERGIC53, is a mannose-specific lectin that recognizes N-linked glycoproteins in the ER lumen (16, 17). The p24 family of proteins function as cargo receptors to regulate ER export of glycosylphosphatidylinositol (GPI)-anchored proteins (18). Mammalian orthologs of yeast ER vesicle (Erv) proteins have also been thought to function as cargo receptors (16). Surfeit locus protein 4 (SURF4), the mammalian ortholog of Erv29p, regulates ER export of soluble proteins, including lipoproteins and proprotein convertase subtilisin/kexin type 9 (PCSK9) (19–21). SURF4 recognizes amino-terminal tripeptide motifs of soluble cargo proteins and participates in ER exit site (ERES) organization (19, 22). The cargo receptors that mediate sorting of Shh in the secretory pathway remain unknown.

Here, we examined trafficking of the N-terminal fragment of Shh without the cholesterol modification (referred to as ShhN). We utilized the Retention Using Selective Hook (RUSH) assay (23) to analyze the kinetics of trafficking of ShhN along the secretory pathway. We reconstituted the packaging of ShhN into transport vesicles in vitro and utilized this assay to quantitatively measure packaging efficiency. Our study reveals cellular factors and underlying mechanisms that mediate the sorting and secretion of Shh, providing insight into the biosynthetic trafficking of Shh.

## Results

### ER Export of ShhN Depends on its Cardin–Weintraub (CW) Motif.

We aimed to determine the mechanisms that mediate secretion of Shh. As a first step, in order to avoid potential complications from posttranslational modification pathways, we examined the N-terminal fragment (ShhN) that lacks the cholesterol modification. A RUSH transport assay (23) was performed to analyze surface delivery in a synchronized manner. In the RUSH assay, HeLa cells were transfected with plasmids encoding mouse N-terminal Shh fragment with the signal peptide removed (amino acid: 25 to 198) fused downstream of enhanced green fluorescent protein (EGFP) and the streptavidin binding peptide (SBP) that had an N-terminal signal peptide derived from IL-2 (SBP-EGFP-ShhN or SBP-EGFP-ShhN^25-198^; *SI Appendix*, Fig. S1*A*). This plasmid also encodes streptavidin fused to a C-terminal ER retention signal (Lys-Asp-Glu-Leu; Str-KDEL). Due to the binding between streptavidin and SBP, SBP-EGFP-ShhN was retained in the ER and colocalized with the ER marker protein disulfide isomerase (PDI) (*SI Appendix*, Fig. S1 *B–E*). When cells are incubated with biotin, SBP is released from streptavidin, thereby releasing SBP-EGFP-ShhN from the ER (*SI Appendix*, Fig. S1 *F*–*I* and *L–M*). At 15 min (*SI Appendix*, Fig. S1 *F–I*) or 30 min (*SI Appendix*, Fig. S1*L*) after biotin treatment, SBP-EGFP-ShhN proteins localized at the juxta-nuclear area in the majority of cells. We counted 100 random cells showing a juxta-nuclear pattern of ShhN 15 min after biotin treatment in three independent experiments, finding that ShhN colocalized with the Golgi marker TGN46 in all of the cells (*SI Appendix*, Fig. S1*J*). Thus, juxta-nuclear ShhN was considered to be located at the Golgi area in the following analyses. SBP-EGFP-ShhN proteins localized at the juxta-nuclear Golgi area in around 80% of cells after 30 min biotin treatment (*SI Appendix*, Fig. S1*L* and quantification in *SI Appendix*, Fig. S1*AF*). A RUSH construct that did not contain the ShhN sequence (SBP-EGFP) was retained in the ER in over 95% of cells after biotin treatment (*SI Appendix*, Fig. S1 *N–O* and quantification in *SI Appendix*, S1*AF*). These results indicate that the RUSH assay is sufficiently robust to analyze the kinetics of ER-to-Golgi transport of ShhN. These results also suggest that ShhN contains motifs that drive efficient export of ShhN from the ER.

**Fig. 1.**
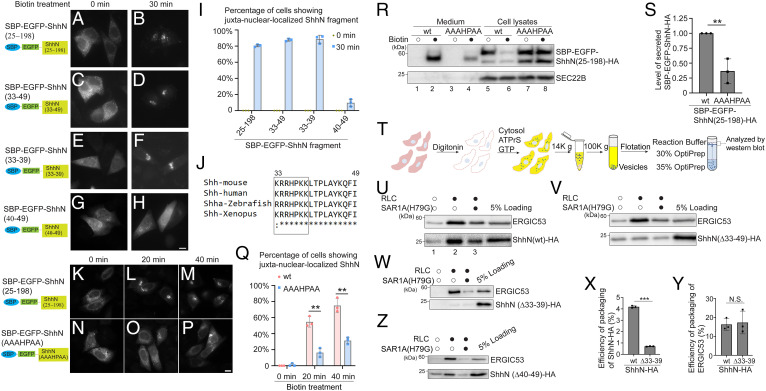
ER-to-Golgi transport of ShhN depends on its KRRHPKK motif. (*A–H* and *K–P*). HeLa cells were transfected with plasmids encoding Str-KDEL and SBP-EGFP-ShhN^25-198^ (*A*, *B*, and *K–M*) or SBP-EGFP–tagged fragments or a mutant version of ShhN (*C–H* and *N*–*P*). Day 1 after transfection, the localization of the different versions of RUSH constructs containing ShhN was analyzed after incubation with biotin for the indicated time (Scale bar, 10 μm). Magnification, 63×. (*I* and *Q*) Quantifications of the percentage of cells showing juxta-nuclear–accumulated EGFP signal after incubation with biotin for the indicated time (mean ± SD; *n* = 3; >100 cells counted for each time point). **P < 0.01. (*J*) Sequence alignment of amino acids 33 to 49 of mouse Shh across species. (*R*) HEK293T cells were transfected with plasmids encoding the indicated constructs. On day 1 after transfection, cells were incubated with biotin for 2 h. After biotin incubation, the level of wild-type (wt) or mutant versions of SBP-EGFP-ShhN^25-198^-HA in the culture medium and in cell lysates was analyzed by immunoblotting using anti-HA and anti-SEC22B antibodies. (*S*) Quantification of the level of secreted SBP-EGFP-ShhN^25-198^-HA normalized to that detected in the wt group (mean ± SD; *n* = 3). In each experimental group, the secreted abundance of ShhN after biotin treatment is normalized to the abundance of ShhN in cell lysates before biotin treatment. **P < 0.01. (*T*) Diagram depicting the vesicle formation assay to reconstitute release of ShhN-HA into transport vesicles. (*U–W* and *Z*) Vesicle formation was performed using the reagents as indicated in cells transfected with plasmids encoding wt or mutant versions of ShhN^1-198^-HA. The vesicle fraction was analyzed by immunoblotting using anti-HA or anti-ERGIC53 antibodies. (*X* and *Y*) Quantification of the percentage of ShhN-HA (*X*) or ERGIC53 (*Y*) that was packaged into transport vesicles (mean ± SD; *n* = 3). ***P < 0.001; N.S., not significant.

We next generated a series of the RUSH constructs containing truncated versions of Shh. We found that SBP-EGFP-ShhN^25-111^, SBP-EGFP-ShhN^25-68^, and SBP-EGFP-ShhN^25-49^ were efficiently exported from the ER to the Golgi (*SI Appendix*, Fig. S1 *Q–S*, *W–Y*, and *AF*). In contrast, SBP-EGFP-ShhN^112-198^, SBP-EGFP-ShhN^69-111^, and SBP-EGFP-ShhN^25-32^ were ER retained (*SI Appendix*, Fig. S1 *T–V, Z–AB*, and *AF*), suggesting that residues 33 to 49 in ShhN are important for ER export (*SI Appendix*, Fig. S1*AG*). Indeed, deleting these residues in ShhN caused defects in ER-to-Golgi transport (*SI Appendix*, Fig. S1 *AC–AE* and *AF*). Interestingly, we found that a RUSH construct composed of Shh residues 33 to 49 fused to SBP-EGFP (SBP-EGFP-Shh^33-49^) was delivered from the ER to the Golgi after biotin treatment with efficiency similar to that of SBP-EGFP-ShhN (Fig. 1 *A–D* and quantification in Fig. 1*I*), indicating that these residues are sufficient to allow SBP-EGFP to exit the ER. Further analysis indicated that residues 33 to 39 in ShhN were sufficient for SBP-EGFP to exit the ER, whereas residues 40 to 49 were not (Fig. 1 *E–H* and quantification in Fig. 1*I*).

The N terminus of Shh is highly conserved. Sequence alignment of this region in mouse ShhN (amino acids 33 to 49) across species revealed a conserved (K/R)RRHPKK motif, termed the CW motif (BBBXXBB, where B represents a basic amino acid) (Fig. 1*J*). The CW motif is predicted to be a heparin binding domain that functions in protein-glycosaminoglycan (GAG) interactions (24). Alanine substitutions of the charged amino acids within this motif caused defects in ER-to-Golgi transport in the RUSH assay (Fig. 1 *K–P* and quantification in Fig. 1*Q*). We therefore asked whether this motif is important for the release of ShhN to the extracellular milieu. To test this, we added an HA tag to the C terminus of SBP-EGFP-ShhN and measured the efficiency of secretion by immunoblotting with antibodies against the HA tag. This analysis indicates that SBP-EGFP-ShhN-HA was secreted into the medium in a biotin-dependent manner (Fig. 1*R*, compare lanes 1 and 2). Mutating the charged amino acids among the CW motif significantly reduced the efficiency of secretion (Fig. 1*R*, compare lanes 2 and 4 and quantification in Fig. 1*S*). These results revealed that the CW motif plays an important role in exporting of ShhN from the ER, eventually to be secreted from the cells. We note that the mobility of SBP-EGFP-ShhN-HA in the medium was slightly different from that in cell lysates (Fig. 1*R*, compare lanes 2 and 5). The change in mobility may be caused by altered posttranslational modifications.

### The CW Motif Is Important for the Packaging of ShhN into COPII Vesicles.

To analyze whether the CW motif is important for the packaging of ShhN into COPII vesicles, we reconstituted vesicular release of ShhN in HEK293T cells (Fig. 1*T*). HEK293T cells transfected with 3xHA-tagged ShhN (ShhN-HA) were permeabilized by digitonin. After permeabilization, the semi-intact cells were washed with buffer to remove endogenous cytosolic proteins. Semi-intact cells were then incubated at 30 °C with rat liver cytosol (RLC), GTP, and an ATP regeneration system (ATPrS) in the presence or absence of a GTP hydrolysis defective mutant form of Sar1A, Sar1A (H79G). The vesicles released were then isolated by centrifugation and analyzed by immunoblotting (Fig. 1*T*). SAR1A (H79G) inhibits COPII-dependent ER export (25) and abolishes the vesicular capture of standard COPII cargo proteins, SEC22B and ERGIC53 (26, 27). In contrast, SAR1A (H79G) does not affect the vesicular release of a *trans* Golgi network (TGN)-derived cargo protein, TGN46, but enhances membrane association of SEC23A/B (28). We propose that GTP hydrolysis of SAR1A allows the release of COPII from vesicle membranes to sustain efficient COPII vesicle formation. Based on these analyses, we consider the dependence on SAR1A (H79G) as indirect evidence for cargo packaging into COPII vesicles. ShhN-HA as well as a COPII cargo protein, ERGIC53, were efficiently packaged into transport vesicles in the presence of cytosol (Fig. 1*U*, compare lanes 1 and 2). The efficiencies of packaging of ShhN-HA and ERGIC53 into transport vesicles were greatly reduced when the vesicle formation assay was performed in the presence of Sar1A (H79G) (Fig. 1*U*, compare lanes 2 and 3), providing evidence that a major fraction of ShhN detected in the vesicle fraction was present in COPII vesicles. Deleting residues 33 to 49 (Fig. 1*V*) or deleting the CW motif (residues 33 to 39) in ShhN caused a significant reduction in the efficiency of packaging of ShhN into vesicles (Fig. 1*W* and quantification in Fig. 1*X*), while the abundance of ERGIC53 in transport vesicles was unchanged (Fig. 1*Y*). Deleting residues 40 to 49 caused no defects (Fig. 1*Z*). These results indicate that the CW motif is important for the packaging of ShhN into COPII vesicles.

### SURF4 Mediates Packaging of ShhN into Transport Vesicles and Regulates the ER-to-Golgi Trafficking and the Secretion of ShhN.

Soluble cargo proteins interact with the cytosolic COPII inner coat indirectly through transmembrane cargo receptors. To reveal cargo receptors that bind ShhN for packaging into transport vesicles, we performed immunoprecipitation experiments. Cell lysates from untransfected HEK293T cells (the Control group) or cells transfected with plasmids encoding HA-tagged ShhN (the ShhN group) or HA-tagged insulin growth factor like-2 (IGF2, the IGF2 group) were incubated with beads conjugated with HA antibodies. The immobilized proteins were then eluted and analyzed by sodium dodecyl sulfate-polyacrylamide gel electrophoresis and Coomassie blue staining (*SI Appendix*, Fig. S2*A*, asterisks indicate the position of ShhN-HA or IGF2-HA). Immunoblot analysis confirmed that IGF2-HA and ShhN-HA were efficiently immunoprecipitated (*SI Appendix*, Fig. S2*B*). The eluted proteins were then trypsin digested and analyzed by mass spectrometry. We identified five proteins in the ShhN-HA group that were not identified in the other two groups (*SI Appendix*, Fig. S2*C*): betaine-homocysteine S-methyltransferase (BHMT), glutamate receptor 1(GRIA1), peptidyl-prolyl cis-trans isomerase B (PPIB), Surfeit locus protein 4 (SURF4), and vacuolar protein sorting–associated protein 51 homolog (VPS51). BHMT is a soluble protein and regulates homocysteine metabolism; GRIA1 belongs to a family of AMPA receptors; PPIB may assist folding of ShhN in the ER; and VPS51 is involved in retrograde transport from early and late endosomes to the TGN and may regulate endosome-to-TGN trafficking of ShhN. SURF4 has been reported to mediate the ER export of soluble proteins, including lipoproteins and proprotein convertase subtilisin/kexin type 9 (PCSK9) (19–21) (*SI Appendix*, Fig. S2*C*, highlighted in red). Therefore, we hypothesized that SURF4 is the cargo receptor for Shh. To verify the mass spectrometry results, cell lysates from HEK293T cells transfected with plasmids encoding Myc-tagged SURF4 (SURF4-Myc) or cells cotransfected with plasmids encoding ShhN-HA and SURF4-Myc or cells cotransfected with IGF2-HA and SURF4-Myc were incubated with beads conjugated with HA antibodies. The immobilized proteins were then analyzed by immunoblot analysis. Coimmunoprecipitation (Co-IP) results indicated that SURF4 interacted with ShhN more robustly than IGF2 (*SI Appendix*, Fig. S2*D*, compare lanes 2 and 3), suggesting that SURF4 may function as a cargo receptor in the ER export of ShhN.

**Fig. 2.**
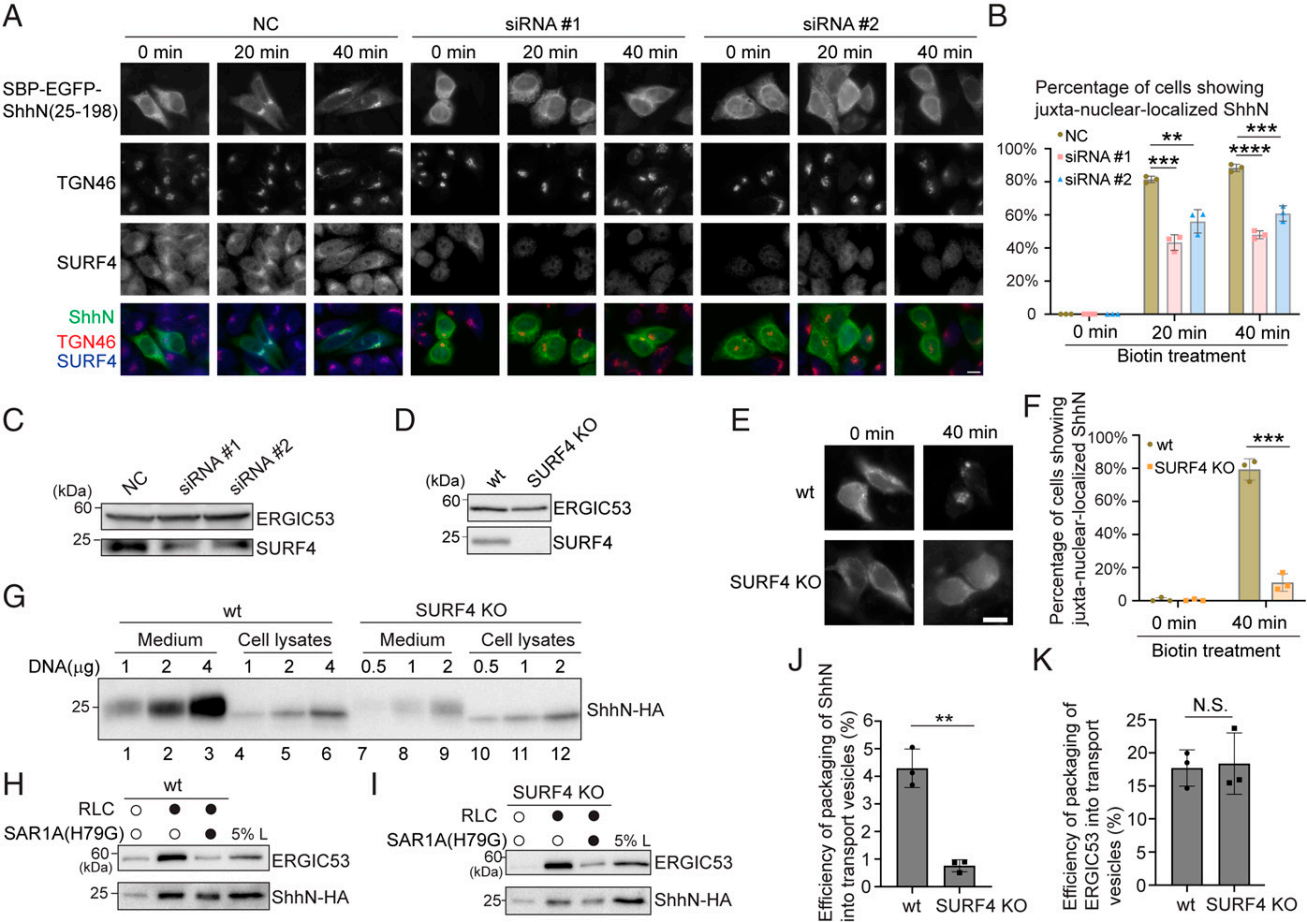
SURF4 mediates packaging of ShhN into transport vesicles and regulates ER-to-Golgi trafficking and the secretion of ShhN. (*A*) HeLa cells were transfected with negative control (NC) siRNA or two different siRNAs against SURF4. At 24 h after transfection, cells were retransfected with plasmids encoding SBP-EGFP-ShhN^25-198^ and Str-KDEL. On day 3 after knockdown, cells were incubated with biotin for the indicated time, and the localization of the indicated proteins was analyzed using antibodies against endogenous TGN46 and SURF4 (Scale bar, 10 μm). Magnification, 63×. (*C* and *D*) The level of SURF4 and ERGIC53 in cell lysates from HeLa cells transfected with NC siRNA or with siRNA against SURF4 (*C*) and from HEK293Trex wild-type (WT) or SURF4 KO cells (*D*) were analyzed by immunoblotting with anti-SURF4 and anit-ERGIC53 antibodies. (*E*) WT or SURF4 KO HEK293Trex cells were transfected with plasmids encoding SBP-EGFP-ShhN^25-198^ and Str-KDEL. On day 3 after knockdown, cells were incubated with biotin for the indicated time, and the localization of ShhN was analyzed (Scale bar, 10 μm). Magnification, 63×. (*B* and *F*) Quantifications of the percentage of cells showing juxta-nuclear–localized SBP-EGFP-ShhN^25-198^ (mean ± SD; *n* = 3; >100 cells counted for each experiment). **P < 0.01; ***P < 0.001; ****P < 0.0001. (*G*) WT or SURF4 KO cells were transfected with the indicated amount of plasmids encoding HA-tagged ShhN^1-198^ (ShhN-HA). Day 1 after transfection, the levels of ShhN-HA in the medium and in cell lysates were analyzed by immunoprecipitation and immunoblotting with anti-HA antibodies. (*H* and *I*) Vesicle formation was performed using the indicated reagents in WT cells (*H*) and SURF4 KO cells (*I*). The vesicle fraction was analyzed by immunoblotting using anti-ERGIC53 or anti-HA antibodies. (*J* and *K*) Quantification of the percentage of ShhN-HA (*J*) or ERGIC53 (*K*) that was packaged into transport vesicles (*n* = 3, mean ± SD). **P < 0.01; N.S., not significant.

We next performed an small interfering RNA (siRNA) knockdown experiment to reduce the expression of SURF4 and analyzed the impact on ER export of ShhN. Nearly all of the cells transfected with siRNA against SURF4 showed reduced SURF4 signal (Fig. 2*A*). Western blot analysis indicates that the level of SURF4 was greatly reduced in the knockdown cells (Fig. 2*C*). The nuclear staining pattern labeled by the SURF4 antibody may be caused by nonspecific staining. We found that knockdown of SURF4 caused a kinetic delay in delivery of SBP-EGFP-ShhN to the Golgi (Fig. 2*A* and quantification in Fig. 2*B*). This defect was rescued by the expression of an siRNA-resistant construct of SURF4 (SURF4^RS^-HA) (*SI Appendix*, Fig. S3 *A–R* and quantification in *SI Appendix*, Fig. S3*S*). Knockdown of SURF4 also caused a kinetic delay in delivery of SBP-EGFP-ShhN^33-39^ to the Golgi (*SI Appendix*, Fig. S4 *A–F* and quantification in *SI Appendix*, Fig. S4*G*). We then generated HEK293Trex SURF4 knockout (KO) cells (Fig. 2*D*). An ER-to-Golgi trafficking defect was also detected in SURF4 KO cells (Fig. 2*E* and quantification in Fig. 2*F*). KO of SURF4 greatly reduced the secretion of ShhN-HA without the SBP-EGFP tag (Fig. 2*G*). Increasing the concentration of plasmids encoding ShhN-HA for transfection caused increased expression levels of ShhN-HA (Fig. 2*G*, compare lanes 4 to 6 and 10 to 12), and the secretion of ShhN-HA in SURF4 KO cells was greatly reduced in each condition (Fig. 2*G*, compares lanes 1 to 3 with lanes 7 to 9). Utilizing the in vitro vesicle formation assay, we found that ShhN-HA is packaged into transport vesicles in a cytosol-dependent manner in HEK293Trex cells, and SAR1A (H79G) reduced the efficiency of packaging (Fig. 2*H*). KO of SURF4 caused a significant reduction of the efficiency of packaging of ShhN into transport vesicles, while the efficiency of packaging of ERGIC53 in transport vesicles was unchanged (Fig. 2 *H–I* and quantifications in Fig. 2 *J–K*).

Since SURF4 binds more efficiently to ShhN than to IGF2 (*SI Appendix*, Fig. S2*D*), we tested whether SURF4 regulates surface delivery of IGF2. We generated a RUSH construct of IGF2 (SBP-EGFP-IGF2). SBP-EGFP-IGF2 was delivered to the Golgi and secreted in a biotin-dependent manner (*SI Appendix*, Fig. S5 *A–C* and *H*, compare lanes 1 and 2). ER-to-Golgi transport and secretion of SBP-EGFP-IGF2 was normal in the SURF4 knockdown cells (*SI Appendix*, Fig. S5 *D–F* and quantifications in *SI Appendix*, Fig. S5 *G* and *H*, compare lanes 2 and 4). These results indicate that SURF4 functions as a cargo receptor to regulate the packaging of ShhN but not IGF2 into COPII vesicles to be delivered to the Golgi.

We next performed a permeabilized cell assay to analyze the colocalization between SURF4 and ShhN at early time points after biotin treatment. HeLa cells expressing SBP-EGFP-ShhN were incubated with biotin for 4 min and permeabilized by digitonin. Subsequently, the semi-intact cells were washed to remove the endogenous cytosolic proteins and then incubated with RLC in the presence of GDP or GTPγS. After such incubation, the COPII components are recruited to punctate structures in the cell periphery (28, 29) and Arf1 is recruited to the juxta-nuclear Golgi area (30) in a GTP-dependent manner. We found that SBP-EGFP-ShhN showed an ER-located pattern after incubation without cytosol and biotin (*SI Appendix*, Fig. S6 *A–C*). When the semi-intact cells were incubated with cytosol, biotin, and GDP, SBP-EGFP-ShhN was partially located in peripheral punctate structures and partially located at the ER in the majority of cells (*SI Appendix*, Fig. S6*D*). When permeabilized cells were incubated with cytosol, biotin, and GTPγS, SBP-EGFP-ShhN was partially located at punctate structures, with the ER pool of ShhN greatly diminished (*SI Appendix*, Fig. S6*G*). Although punctate structures of ShhN were detected in the cell periphery after incubation in the presence of GDP or GTPγS, we did not detect accumulation of ShhN at the juxta-nuclear Golgi area 15 min after incubation with cytosol and nucleotides (*SI Appendix*, Fig. S6 *D* and *G*). These analyses indicate that this assay locks the ER export process at the cargo sorting stage, providing a convenient way to accumulate cargo proteins at ERES and to analyze the colocalization between cargo proteins and their receptors.

Many of the punctate structures of SBP-EGFP-ShhN colocalized with SURF4 in the presence of either GDP or GTPγS (*SI Appendix*, Fig. S6 *D–I*, magnified views in *SI Appendix*, Fig. S6 *F′*–*I′′′*), suggesting that SURF4 is associated with ShhN in a GTP-independent manner. SEC31A was recruited to the semi-intact cells in a GTP-dependent manner (*SI Appendix*, Fig. S6 *K* and *N*), consistent with previous reports (28, 29). Many ShhN punctate structures partially overlapped with SEC31A in the presence of GTPγS (*SI Appendix*, Fig. S6 *M–O*, magnified views in *SI Appendix*, Fig. S6 *O′*–*P′′′*), further suggesting stalled transport intermediates.

### SURF4 Directly Interacts with the CW Motif on ShhN at the ER.

**Fig. 3.**
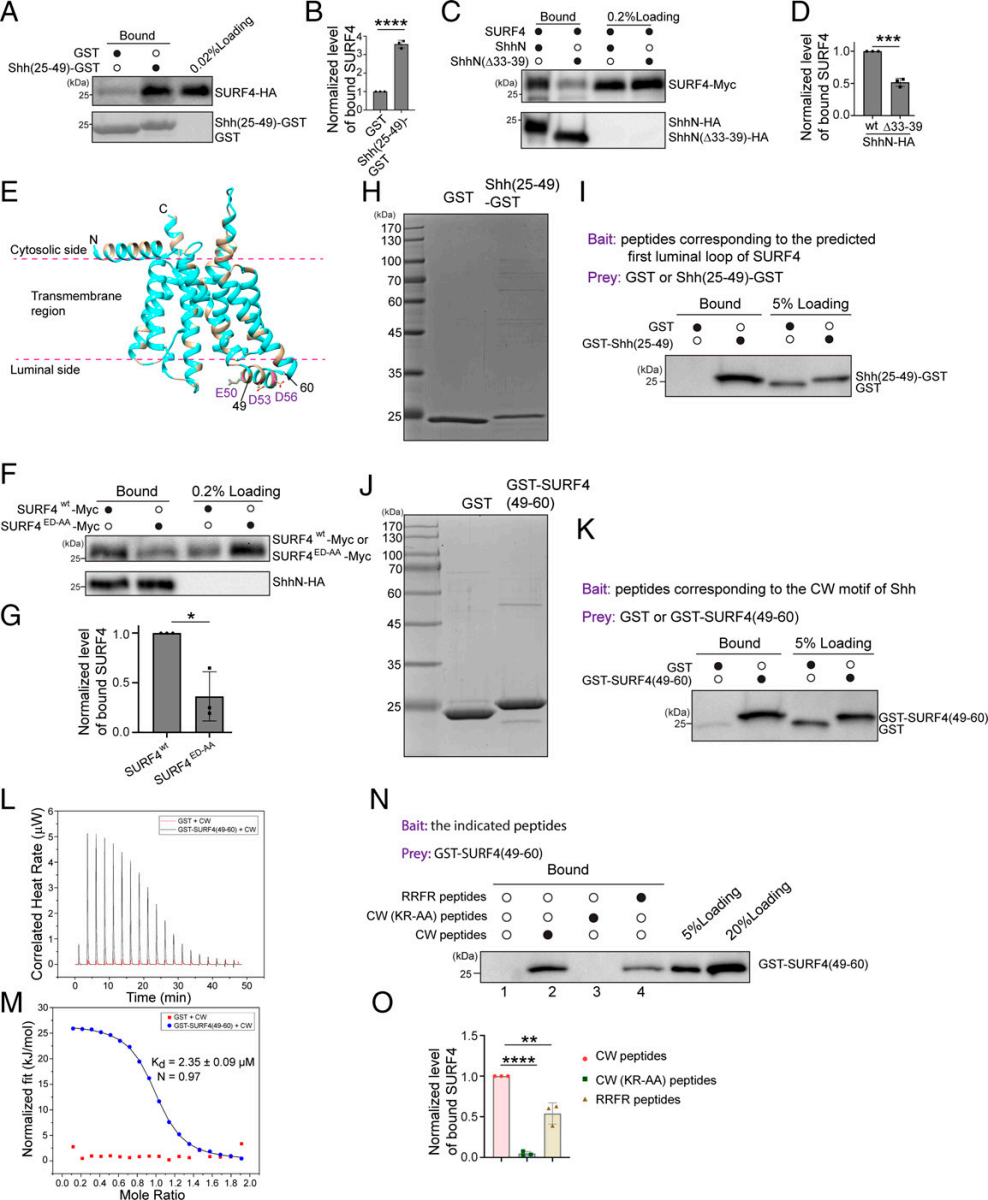
The CW motif of ShhN directly interacts with the predicted first luminal loop of SURF4. (*A*) Purified GST or GST-tagged human ShhN^25-49^ was incubated with lysates from HEK293T cells transfected with SURF4-HA. After incubation, the bound proteins were analyzed by immunoblotting with anti-HA antibodies. (*B*) Relative levels of SURF4-HA that bound to GST or ShhN^25-49^-GST were quantified (*n* = 3, mean ± SD). The level of SURF4-HA that bound to GST or ShhN^25-49^-GST was normalized to the corresponding bait protein, and this value was then normalized to the level of SURF4-HA that bound to GST in each experimental group. ****P < 0.0001. (*C*) HEK293T cells were cotransfected with plasmids encoding the indicated constructs. Day 1 after transfection, cells were treated in 2 mM dithiobis(succinimidyl propionate) (DSP), and cell lysates were incubated with beads conjugated with HA antibodies. The bound proteins were analyzed by immunoblotting with anti-HA or anti-Myc antibodies. (*D*) The percentage of SURF4 that bound to ShhN^Δ33-39^-HA was normalized to that bound to ShhN-HA. The normalized abundance was then quantified (*n* = 3, mean ± SD). ***P < 0.001. (*E*) The structure of SURF4 predicted by AlphaFold. Hydrophobic amino acids are highlighted in light blue. (*F*) Co-IP was performed in HEK293T cells expressing the indicated constructs in the presence of DSP. The bound proteins were analyzed by immunoblotting with anti-HA or anti-Myc antibodies. (*G*) The percentage of SURF4^ED-AA^-Myc that bound to ShhN-HA was normalized to the percentage of SURF4^WT^-Myc. The normalized abundance was then quantified (*n* = 3, mean ± SD). *P < 0.05. (*H* and *J*) GST, Shh^25-49^-GST, GST-SURF4^49-60^ were purified from *Escherichia coli* and analyzed by commassie blue staining. (*I* and *K*). CW or SURF4-luminal peptides were covalently linked to thiopyridone Sepharose 6B and incubated with the indicated prey proteins. After incubation, the bound proteins were analyzed by immunoblotting with anti-GST antibodies. (*L* and *M*) Isothermal titration calorimetry–based measurement of the interaction between CW peptides and GST-SURF4^49-60^ or GST. (*N*) CW, CW(KR-AA), or RRFR peptides were covalently linked to thiopyridone Sepharose 6B, incubated with GST-SURF4^49-60^. After incubation, the bound proteins were analyzed by immunoblotting with anti-GST antibodies. (*O*) Levels of GST-SURF4^49-60^ bound to the indicated peptides were quantified (*n* = 3, mean ± SD). The quantification is normalized to the level of GST-SURF4^49-60^ that bound to CW peptides in each experimental group. **P < 0.01; ****P < 0.0001.

Since ER export of ShhN depends on its CW motif (Fig. 1), we next tested whether this signal mediates interaction with SURF4. Purified GST-tagged ShhN^25-49^, which contains the CW motif in an N-terminal orientation, interacted with SURF4-HA from lysates of HEK293T cells overexpressing SURF4-HA. In contrast, GST alone recruited SURF4-HA poorly (Fig. 3*A* and quantification in Fig. 3*B*). To test whether the CW motif is important for the interaction between SURF4 and ShhN, we performed co-IP experiments using HEK293T cells cotransfected with plasmids encoding SURF4-Myc and ShhN-HA or HA-tagged CW motif–depleted ShhN (ShhN^Δ33-39^-HA). We found that the percentage of SURF4-Myc that bound to ShhN-HA was significantly higher than the percentage of SURF4-Myc that bound to ShhN^Δ33-39^-HA (Fig. 3*C* and quantification in Fig. 3*D*), suggesting that ShhN interacted with SURF4 through the CW motif.

The N and C termini of SURF4 are thought to be exposed to the cytosolic face of the ER, similar to the yeast homolog of SURF4, Erv29 (21, 31, 32). The structure of human SURF4 predicted by AlphaFold (33, 34) indicates that SURF4 contains eight transmembrane helixes (Fig. 3*E*, hydrophobic amino acids highlighted in light blue). The cytosolic N terminus of SURF4 is predicted to form an amphipathic helix with the hydrophobic side contacting the cytosolic leaflet of the lipid bilayer. Interestingly, the first luminal loop of SURF4 (amino acid: 49 to 60) is predicted to form a helix with three negatively charged residues that point toward the lumen (residues E50, D53, and D56 highlighted in Fig. 3*E*). Co-IP analysis indicates that mutating these residues to alanine significantly reduced the interaction between SURF4-Myc and ShhN-HA (Fig. 3 *F–G*).

To measure a direct interaction, we immobilized the first luminal loop of SURF4 (SEQRDYIDTTWNC, referred to as SURF4 luminal peptides) on beads and then performed pull down analysis using purified GST and Shh^25-49^-GST as prey (Fig. 3*H*). Strikingly, we found that Shh^25-49^-GST but not GST interacts with SURF4 luminal peptides (Fig. 3*I*). As an additional test, we performed pull down analysis using peptides corresponding to the CW motif (KRRHPKKC, referred to as CW peptides) as bait and purified GST-SURF4^49-60^ as prey (Fig. 3*J*). We found that GST-SURF4^49-60^ binds CW peptides, whereas GST binds weakly (Fig. 3*K*). Isothermal titration calorimetry–based measurement indicates that GST-SURF4^49-60^ bound to CW peptides with a K_d_ of 2.35 ± 0.09 μM, whereas no binding was detected between GST and CW peptides (Fig. 3 *L* and *M*). Further analysis indicates that GST-SURF4^49-60^ bound more efficiently to the wild-type CW sequence than an alanine substituted mutant (AAAHPAAC, referred to as CW(KR-AA) peptides) (Fig. 3*N*, compare lanes 2 and 3, and quantification in Fig. 3*O*). These results indicate that the CW motif directly interacts with the first luminal domain of SURF4 through electrostatic interactions. We then immobilized peptides corresponding to the first intracellular loop of Frizzled6 (VRRFRYPERPC, referred to as RRFR) (27) on beads. Although RRFR peptides are also positively charged, the level of GST-SURF4^49-60^ bound to RRFR peptides was significantly lower than that bound to the CW peptides (Fig. 3*N*, compare lanes 2 and 4, and quantification in Fig. 3*O*).

SURF4 is shown to localize to the ER, ERES, and ER-Golgi intermediate compartment (ERGIC) (20, 22). Mutations in the COPI-binding motif of SURF4 or expression of the GTPase-defective mutant form of Arf1, Arf1(Q71L), accumulate SURF4 at the Golgi, suggesting that SURF4 cycles between the ER and the Golgi (21). When SURF4-HA was coexpressed with SBP-EGFP-ShhN, it was located at the ER in the absence of biotin in ∼60% of the SURF4- and ShhN-coexpressing cells (*SI Appendix*, Fig. S7 *A–C* and quantification in *SI Appendix*, Fig. S7*J*). At 20 min after biotin treatment, SBP-EGFP-ShhN was located at the juxta-nuclear Golgi area (*SI Appendix*, Fig. S7 *D–F*). Interestingly, SURF4-HA was colocalized with SBP-EGFP-ShhN at the juxta-nuclear Golgi area 20 min after biotin treatment in ∼90% of the SURF4- and ShhN-coexpressing cells (*SI Appendix*, Fig. S7 *D–F* and quantification in *SI Appendix*, Fig. S7*J*). Quantification indicates that the fraction of cells showing juxta-nuclear SURF4-HA was significantly increased 20 min after biotin treatment in the coexpressing cells (*SI Appendix*, Fig. S7*J*). At 60 min after biotin treatment, SBP-EGFP-ShhN was exported out of the Golgi and was located to some intracellular punctate structures in the cytoplasm and near the cell surface (*SI Appendix*, Fig. S7*G*). In contrast, SURF4-HA was localized in the ER and the Golgi area at this time point (*SI Appendix*, Fig. S7*H*). These analyses indicate that SURF4 traffics together with SBP-EGFP-ShhN from the ER to the Golgi, then SURF4 is retrieved to the ER while ShhN travels to the cell surface.

As an additional experiment to test whether SURF4 traffics together with ShhN to the Golgi, we compared the localization of SURF4 20 min after biotin treatment with the localization of a *cis*-Golgi marker, GM130, or with the localization of an ERGIC marker, ERGIC53, in HeLa cells. GM130 showed a juxta-nuclear localization pattern (*SI Appendix*, Fig. S7*K*). ERGIC53 showed a punctate localization pattern in the cell periphery and also a juxta-nuclear localization pattern (*SI Appendix*, Fig. S7*L*). The punctate pattern of ERGIC53 is adjacent to the ERES (35), and the juxta-nuclear pattern of ERGIC53 partially colocalized with GM130 (*SI Appendix*, Fig. S7*M*). The majority of SURF4-HA was located at the juxta-nuclear area 20 min after biotin treatment (*SI Appendix*, Fig. S7 *P* and *T*). The juxta-nuclear–located SURF4 overlapped more with GM130 than with ERGIC53 (*SI Appendix*, Fig. S7 *R* and *V*). We quantified the colocalization between ERGIC53 and GM130 in the juxta-nuclear area labeled by GM130 (*SI Appendix*, Fig. S7*N*). We also quantified the colocalization between SURF4-HA and GM130 or ERGIC53 in the juxta-nuclear area labeled by SBP-EGFP-ShhN (*SI Appendix*, Fig. S7*W*). The Pearson correlation coefficient (Pearson’s R value) was calculated as an indicator of the colocalization. This quantification indicates that the colocalization between SURF4-HA and GM130 was significantly higher than that between SURF4-HA and ERGIC53 (*SI Appendix*, Fig. S7*W*, R values of 0.714 and 0.496, respectively). These analyses suggest that SURF4 and ShhN traffic together to the *cis* Golgi 20 min after biotin treatment.

We hypothesized that SURF4 interacts with ShhN at the ER to enrich COPII vesicles with ShhN, and after delivery to the Golgi, SURF4 dissociates from ShhN to be retrieved to the ER. To test this hypothesis, we performed co-IP experiments using HEK293T cells cotransfected with plasmids encoding SURF4-Myc and Str-KDEL_SBP-EGFP-ShhN-HA with or without biotin treatment. Under the no-biotin condition, the majority of SURF4 and ShhN locate to the ER (*SI Appendix*, Fig. S7 *A–C*) and robustly co-IP (*SI Appendix*, Fig. S7 *X* and *Y*). Under conditions of biotin treatment, combined with incubation at 20 °C to block cargo export from the TGN, co-IP was reduced (*SI Appendix*, Fig. S7 *X* and *Y*). Together, these data suggest that ShhN and SURF4 interact with each other at the ER and separate from each other after entering the Golgi. 

### Proteoglycans (PGs) Regulate Export of ShhN out of the TGN.

PGs are important for TGN export of soluble cargo proteins including the soluble enzyme lipoprotein lipase (LPL) (36). Therefore, we tested whether PGs regulate TGN export of ShhN. We treated the cells with xyloside, which inhibits the attachment of GAGs during PG maturation. Treatment with xyloside did not cause defects in ER-to-Golgi transport of SBP-EGFP-ShhN (*SI Appendix*, Fig. S8 *A–E*). We then analyzed the kinetics of TGN export of ShhN using the RUSH assay. HeLa cells expressing Str-KDEL and SBP-EGFP-ShhN were incubated at 20 °C in the presence of biotin to accumulate cargo in the TGN, then shifted to 32 °C to release cargo. After the 20 °C incubation, SBP-EGFP-ShhN accumulated at the juxta-nuclear Golgi area with no detectable punctate structures in the cytoplasm (*SI Appendix*, Fig. S9 *A*–*F*, and magnified views in *SI Appendix*, Fig. S9 *A′* and *D′*). At 45 min after incubation at 32 °C, SBP-EGFP-ShhN in the majority of cells showed a punctate localization pattern (*SI Appendix*, Fig. S9 *G*–*I*, and magnified view in *SI Appendix*, Fig. S9*G′*). We hypothesize that these punctate structures are TGN-derived transport vesicles enriched with SBP-EGFP-ShhN. The average number of punctate structures containing SBP-EGFP-ShhN in each expressing cell 45 min after incubation at 32 °C was significantly decreased after xyloside treatment (*SI Appendix*, Fig. S9 *J*–*L*, magnified views in *SI Appendix*, Fig. S9*J′* and quanitification in *SI Appendix*, Fig. S9*M*).

**Fig. 4.**
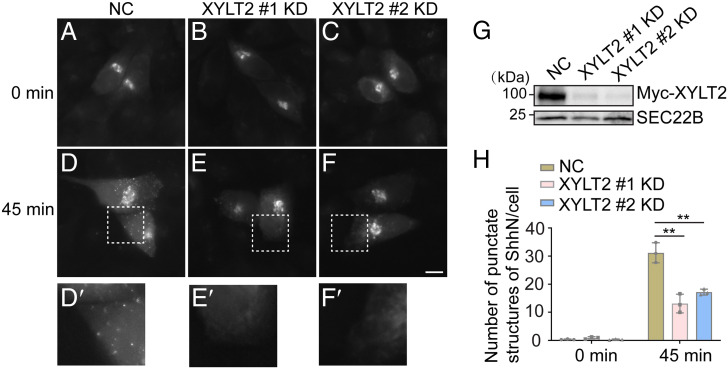
Synthesis of PGs regulates export of ShhN out of the TGN. (*A–F*) HeLa cells were transfected with NC siRNA or two different siRNAs against XYLT2. At 48 h after transfection, cells were retransfected with plasmids encoding Str-KDEL and SBP-EGFP-ShhN^25-198^. On day 3 after knockdown, cells were treated with biotin and incubated in the 20 °C for 2 h. Then the cells were incubated at 32 °C for 0 or 45 min, and the localization of Shh was analyzed (Scale bar, 10 μm). Magnification, 63× . The magnified views of the indicated area in panels *D–F* are shown in panels *D′*–*F′*. (*G*) HEK293T were transfected with negative control (NC) siRNA or siRNA against XYLT2. At 48 h after transfection, cells were retransfected with plasmids encoding Myc-XYLT2. On day 3 after knockdown, the level of SEC22B and Myc-XYLT2 in cell lysates was analyzed by immunoblotting using anti-Myc or anti-SEC22B antibodies. (*H*) Quantifications of the number of punctate structures containing SBP-EGFP-ShhN^25-198^ per cell at different time points after biotin treatment (*n* = 3, mean ± SD, over 20 cells were quantified in each experimental group). **P < 0.01.

We next performed a live imaging analysis to visualize the surface delivery of SBP-EGFP-ShhN. We found that SBP-EGFP-ShhN was delivered to the juxta-nuclear Golgi area after biotin treatment (*SI Appendix*, Fig. S10 *A*–*H* and Movies S1 and S2). We observed punctate structures of SBP-EGFP-ShhN in the cytoplasm during post-Golgi trafficking of SBP-EGFP-ShhN, and the majority of these punctate structures showed a clear mobility toward the cell surface (*SI Appendix*, Fig. S10 *C*, *D*, *G*, and *H*, and magnified views in *SI Appendix*, Fig. S10 *C′*–*D′′* and *G′*–*H′′*, and Movies S1 and S2). These observations are consistent with our hypothesis, suggesting that these punctate structures are post-Golgi vesicles. Xyloside treatment did not block the ER-to-Golgi transport of SBP-EGFP-ShhN (*SI Appendix*, Fig. S10 *I*, *J*, *M*, and *N*, and Movies S3 and S4), but the number of punctate structures during post-Golgi trafficking was greatly reduced compared to the cells without drug treatment (*SI Appendix*, Fig. S10 *K*, *L*, *O*, and *P* and magnified views in *SI Appendix*, Fig. S10 *K′*–*L′′* and *O′*–*P′′*, quantification in *SI Appendix*, Fig. S10 *Q* and *R*). We did not observe any apparent movement of the punctate structures toward the cell surface in the drug-treated cells (Movies S3 and S4). The live imaging analyses were consistent with our analyses using fixed cells, indicating that synthesis of PGs regulates export of ShhN out of the TGN.

As an additional experiment to test the effect of PG synthesis on TGN export of ShhN, we knocked down expression of xylosyltransferase 2 (XYLT2), which catalyses the attachment of GAG chains to PG core proteins, using two different siRNAs (Fig. 4*G*). We then performed a temperature shift experiment, in which cells were incubated at 20 °C in the presence of biotin to accumulate cargo in the TGN, then shifted to 32 °C to release cargo. We quantified the number of punctate structures of SBP-EGFP-ShhN 45 min after incubation at 32 °C. The average number of punctate structures of SBP-EGFP-ShhN in each expressing cell was significantly decreased in cells transfected with either siRNA against XYLT2 (Fig. 4 *A*–*F*, magnified views in Fig. 4 *D*′–*F*′ and quantification in Fig. 4*H*), suggesting a defect in export of SBP-EGFP-ShhN out of the TGN in XYLT2 knockdown cells.

### PGs Compete with SURF4 to Bind ShhN and Facilitate Trafficking of ShhN through the Golgi.

**Fig. 5.**
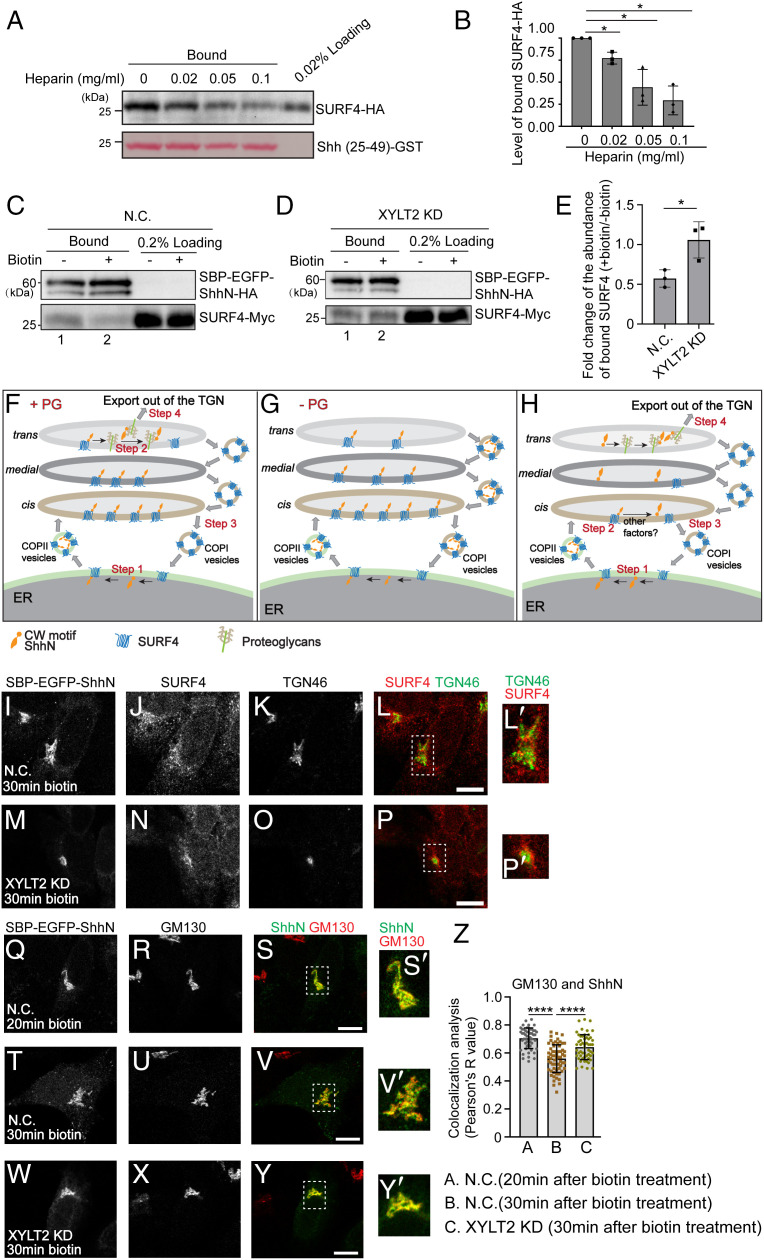
PGs compete with SURF4 to bind ShhN and facilitate trafficking of ShhN through the Golgi. (*A*) Purified GST-tagged human ShhN^25-49^ was incubated with lysates from HEK293T cells transfected with SURF4-HA in the presence of the indicated concentrations of heparin. After incubation, the bound proteins were analyzed by immunoblotting with anti-HA antibodies. (*B*) Relative levels of SURF4-HA that bound to ShhN^25-49^-GST were quantified (*n* = 3, mean ± SD). The quantification is normalized to the level of SURF4-HA that bound to ShhN^25-49^-GST in the absence of heparin in each experimental group. *P < 0.05. (*C* and *D*) HEK293T cells were transfected with negative control (NC) siRNA or siRNAs against XYLT2. At 48 h after transfection, cells were retransfected with plasmids encoding SURF4-Myc and SBP-EGFP-ShhN^25-198^-HA (referred to as SBP-EGFP-ShhN-HA). On day 3 after knockdown, cells were incubated at 20 °C for 2 h in the absence or presence of biotin. Then, cells were treated with 2 mM DSP, and the cell lysates were incubated with beads conjugated with HA antibodies. The bound proteins were analyzed by immunoblotting with anti-HA or anti-Myc antibodies. (*E*) Relative levels of SURF4-Myc that bound to SBP-EGFP-ShhN-HA were quantified (*n* = 3, mean ± SD). In each experimental group, the levels of bound SURF4-Myc after biotin treatment is normalized to the levels of bound SURF4-Myc before biotin treatment.*P < 0.05. (*F–H*) Our proposed model depicting the molecular mechanisms regulating sorting and secretion of ShhN. (*I–Y*) HeLa cells transfected with negative control siRNA (NC) or siRNA against XYLT2 (XYLT2 KD) were treated with biotin for 20 or 30 min, and the localizations of the indicated proteins were analyzed (Scale bar, 10 μm). Magnification, 63×. The magnified views of the indicated area in panels *L, P, S, V,* and *Y* were shown in panels *L′, P′, S′, V′,* and *Y′*. (*Z*) Quantifications of the colocalization between SBP-EGFP-ShhN and GM130 in the juxta-nuclear area labeled by SBP-EGFP-ShhN (mean ± SD, each dot represents one cell). ****P < 0.0001.

The CW motif of Shh interacts with GAG chains of PGs (37, 38). Mutating this motif causes defects in Hh signaling in mice (37, 39). Using GST pull downs, we found that the addition of a GAG, heparin, inhibited the interaction between ShhN and SURF4 in a concentration-dependent manner (Fig. 5*A* and quantification in Fig. 5*B*), suggesting heparin competes with SURF4 to bind ShhN. As GAG chains are attached to the PG core proteins in the Golgi, we hypothesized that this competition mediates the dissociation of SURF4 from ShhN at the Golgi. To test this hypothesis, we performed crosslinking co-IP experiments in the absence (ER-localized complex) and presence (Golgi-localized complex) of biotin. Precipitation of SURF4-Myc with the Golgi-localized ShhN RUSH construct was reduced relative to that coprecipitated in the ER-localized condition (Fig. 5*C*). In contrast, in XYLT2 knockdown cells, the abundance of SURF4 that associated with the Golgi-localized SBP-EGFP-ShhN was equivalent to that coprecipitating with the ER-localized pool (Fig. 5*D* and quantification in Fig. 5*E*). These analyses suggest that blocking PG synthesis causes defects in the dissociation of SURF4 from the ShhN at the Golgi.

We propose that the SURF4-ShhN complex, after being delivered to the Golgi, will dissociate via a competitive interaction with PGs. SURF4 would then be retrieved back to the ER via COPI vesicles, and ShhN in association with PGs would be exported toward the cell surface (Fig. 5*F*). This model predicts that defects in the dissociation of SURF4 from ShhN at the Golgi may result in two possible consequences: 1) SURF4 penetrates to the TGN area in XYLT2 knockdown cells after biotin treatment or 2) accumulation of ShhN at the *cis* Golgi together with its associated SURF4 (Fig. 5*G*). To test the first possibility, we analyzed the colocalization between endogenous SURF4 and TGN46 in XYLT2 KD cells 30 min after biotin treatment. However, we did not detect a clear colocalization between SURF4 and TGN46 in control cells or in the knockdown cells (Fig. 5 *I–P*, magnified views in Fig. 5 *L*′ and *P*′). One explanation is that SURF4 is rapidly and constitutively retrieved back to the *cis* Golgi after the SURF4-ShhN complex is delivered to the Golgi, making TGN-located SURF4 difficult to detect.

To test the second possibility, we analyzed trafficking of SBP-EGFP-ShhN through the *cis* Golgi in cells treated with biotin for 20 or 30 min. We quantified the colocalization between SBP-EGFP-ShhN and GM130 in the juxta-nuclear area labeled by SBP-EGFP-ShhN. We found that the colocalization between GM130 and the juxta-nuclear-located SBP-EGFP-ShhN was significantly reduced in cells treated with biotin for 30 min than in cells treated with biotin for 20 min (Fig. 5 *Q–V*, magnified views in Fig. 5 *S′* and *V′*, quantifications in Fig. 5*Z*), demonstrating the passage of SBP-EGFP-ShhN out of the *cis* Golgi during biotin treatment. In XYLY2 KD cells, we found that colocalization between SBP-EGFP-ShhN and GM130 was significantly higher than that in control cells 30 min after biotin treatment (Fig. 5 *T–Y*, magnified views in Fig. 5 *V′* and *Y′*, quantifications in Fig. 5*Z*). This analysis suggests that blocking PG synthesis causes defects in trafficking of ShhN beyond the *cis* Golgi.

The protein interaction and colocalization analyses provide evidence suggesting that displacement of SURF4 from ShhN by PGs is important for trafficking of ShhN through the Golgi. In addition to this mechanism, many soluble cargo proteins are dissociated from their receptors at low pH (40, 41). The luminal pH of the ER is nearly neutral, and the luminal pH of the TGN is around 6.0 (42). We found that lowering the pH from 7.2 to 6.0 did not cause a significant reduction of the abundance of SURF4-HA that bound Shh^25-49^-GST (*SI Appendix*, Fig. S11*A*, compare lanes 1 and 3, quantification in *SI Appendix*, Fig. S11*B*), suggesting the slightly acidic pH at the TGN is unlikely to drive release of SURF4 from ShhN. At pH 6.0, heparin still inhibits the ShhN-SURF4 interaction (*SI Appendix*, Fig. S11 *C* and *D*), indicating that PGs can compete with SURF4 to bind ShhN in the slightly acidic environment at the TGN. However, we cannot rule out the possibility that other molecules also contribute to release of SURF4 from ShhN at the Golgi, with the released ShhN subsequently engaging with PGs at the TGN for onward traffic (Fig. 5*H*). The analyses here also indicate that delays in intra-Golgi transport of ShhN in XYLT2 knockdown cells may indirectly interfere with TGN export.

### SURF4 and Synthesis of PGs Are Important for ER Export and TGN Export of Full-Length Shh, Respectively.

Since the ShhN construct we used is not modified by cholesterol, we wanted to test the effects of SURF4 under more native conditions. We therefore generated a RUSH construct of full-length Shh (SBP-EGFP-Shh^FL^). To test whether the proteins encoded by the RUSH construct can be processed into the N- and C-terminal fragments, HEK293T cells were transfected with the RUSH construct of Shh^FL^ bearing an N- or C-terminal HA tag (SBP-EGFP-HA-Shh^FL^ or SBP-EGFP-Shh^FL^-HA). Immunoblot analyses showed that two bands can be detected by anti-HA antibody in cell lysates from HEK293T cells expressing SBP-EGFP-HA-Shh^FL^ (HA-Shh^FL^). Their molecular weights matched those predicted for the N-terminal fragment and full-length precursor of Shh, SBP-EGFP-HA-ShhN (∼54 kDa), and SBP-EGFP-HA-Shh^FL^ (∼80 kDa) (*SI Appendix*, Fig. S12*A*, lane 1). Two major bands can be detected by anti-HA antibody in cell lysates from HEK293T cells expressing SBP-EGFP-Shh^FL^-HA (Shh^FL^-HA). The molecular weights matched the predicted C-terminal fragment and full-length precursor of Shh, ShhC-HA (∼33 kDa), and SBP-EGFP-Shh^FL^-HA (∼80 kDa) (*SI Appendix*, Fig. S12*A*, lane 2). These analyses indicate that SBP-EGFP-Shh^FL^ is processed into N- and C-terminal fragments. SBP-EGFP-Shh^FL^ was not detected in the media fraction from cell cultures treated with biotin (*SI Appendix*, Fig. S12*B*)*.* We suggest that the N-terminal fragment generated by cleavage of SBP-EGFP-HA-Shh^FL^ is modified by cholesterol, and this modification prevents the release of the N-terminal fragment from the plasma membrane to the medium. We also detected a reduction in the abundance of the processed RUSH fusion of Shh^FL^ in cell lysates after biotin treatment (*SI Appendix*, compare lanes 3 and 4 in *SI Appendix*, Fig. S12*B*).  We hypothesize that some of the N-terminal fragment of Shh, after delivery to the cell surface, is internalized and routed to lysosomes for degradation. We therefore performed immunofluorescence to visualize the localization of processed Shh fragments in HeLa cells expressing SBP-EGFP-Shh^FL^-HA. The processed N-terminal fragment of Shh was annotated as SBP-EGFP-ShhN, and the processed C-terminal domain of Shh was annotated as ShhC-HA. SBP-EGFP-ShhN and ShhC-HA were located at the ER in the absence of biotin (*SI Appendix*, Fig. S12 *C*–*E*). At 20 min after biotin treatment, ∼80% cells showed Golgi-localized SBP-EGFP-ShhN, whereas ShhC-HA was still in the ER (*SI Appendix*, Fig. S12 *F*–*H* and quantification in *SI Appendix*, Fig. S12*I*). These results indicate that the N-terminal fragment of SBP-EGFP-Shh^FL^ can be transported from the ER to the Golgi, while the C-terminal fragment cannot.

We next used the RUSH constructs of Shh^FL^ to analyze ER export and TGN export. We found that SBP-EGFP-Shh^FL^ can be delivered to the juxta-nuclear Golgi area in a biotin-dependent manner (*SI Appendix*, Fig. S13 *A*–*F*). The kinetics of ER-to-Golgi transport of SBP-EGFP-Shh^FL^ was significantly reduced in SURF4 knockdown cells, and this defect was rescued by expressing siRNA-resistant SURF4-HA (*SI Appendix*, Fig. S13 *G*–*R* and quantification in *SI Appendix*, Fig. S13*AB*). ER-to-Golgi trafficking defects were also observed in SURF4 KO cells (*SI Appendix*, Fig. S13 *S*–*X* and quantification in *SI Appendix*, Fig. S13*AC*), and this defect was rescued by expressing the siRNA-resistant SURF4-HA (*SI Appendix*, Fig. S13 *Y*–*AA* and quantification in *SI Appendix*, Fig. S13*AC*).

**Fig. 6.**
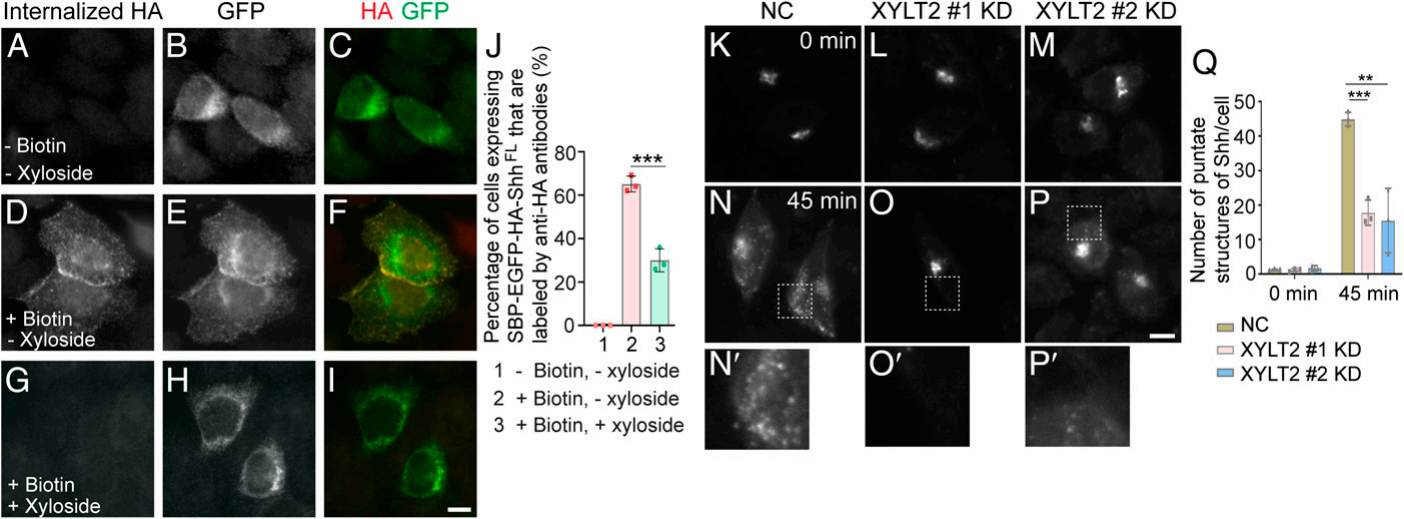
Synthesis of PGs regulates TGN-to-cell surface delivery of SBP-EGFP-Shh^FL^. (*A–I*) HeLa cells were untreated (*A–F*) or treated (*G–I*) with 2.5 mM xyloside. At 24 h after xyloside treatment, cells were transfected with plasmids encoding Str-KDEL_SBP-EGFP-HA-Shh^FL^. At 48 h after xyloside treatment, cells were treated without biotin (*A–C*) or with biotin for 1 h (*D–I*). Then, the antibody uptake assay was performed using mouse anti-HA antibodies. The surface and total SBP-EGFP-HA-Shh^FL^ were detected by immunofluorescence (Scale bar, 10 μm). Magnification, 63×. (*J*) Quantification of the percentage of cells showing surface-localized Shh (mean ± SD; *n* = 3; >50 cells counted for each group). ***P < 0.001. (*K–P′*) HeLa cells were transfected with NC siRNA or two different siRNAs against XYLT2. At 48 hr after transfection, cells were retransfected with plasmids encoding Str-KDEL and SBP-EGFP-Shh^FL^. On day 3 after knockdown, cells were treated with biotin and incubated at 20 °C for 2 h. Cells were then incubated at 32 °C for 0 or 45 min, and the localizations of Shh constructs were analyzed (Scale bar, 10 μm). Magnification, 63× . The magnified views of the indicated areas in panels *N*–*P* are shown in panels *N′*–*P′*. (*Q*) Quantifications of the number of punctate structures containing SBP-EGFP-Shh^FL^ per cell at different time points after biotin treatment (*n* = 3, mean ± SD, over 20 cells were quantified in each experimental group). **P < 0.01; ***P < 0.001.

SBP-EGFP-Shh^FL^ is not clearly detectable on the cell surface after biotin treatment, presumably because the membrane-anchored protein is rapidly internalized after delivery to the plasma membrane. To test whether synthesis of PG is important for surface delivery of Shh, we utilized the RUSH construct of Shh that contains an HA tag (SBP-EGFP-HA-Shh^FL^) and performed antibody uptake experiments. Mouse anti-HA antibodies were used to label SBP-EGFP-HA-Shh^FL^ that had been delivered to the cell surface, and rabbit anti-GFP antibodies were used to label the total signal of SBP-EGFP-HA-Shh^FL^. The Shh-expressing cells were not detected by mouse anti-HA antibodies in the absence of biotin (Fig. 6 *A–C* and *J*). At 1 h after biotin treatment, around 65% of the Shh-expressing cells were detected by anti-HA antibodies in the absence of xyloside (Fig. 6 *D–F*, and *J*). In contrast, the percentage of Shh-expressing cells detected by anti-HA antibodies was significantly lower in the presence of xyloside after biotin treatment (65 versus 30%, Fig. 6 *G–I*, and *J*). This result indicates that blocking PG synthesis causes defects in surface delivery of SBP-EGFP-HA-Shh^FL^. SBP-EGFP-HA-Shh^FL^ showed a juxta-nuclear-located pattern and ER-like pattern in many of the cells treated with xyloside after biotin treatment (Fig. 6*H*). The continued localization of SBP-EGFP-HA-Shh^FL^ at the ER after biotin treatment may be caused by incomplete cycloheximide effectiveness or by inappropriate Golgi-ER retrieval of the SURF4-Shh complex in xyloside-treated cells.

To demonstrate that xyolside treatment does not cause global secretory defects, we analyzed trafficking of a RUSH construct of E-cadherin (SBP-EGFP-E-cadherin). After 20 °C incubation, SBP-EGFP-E-cadherin accumulated at the juxta-nuclear Golgi area (*SI Appendix*, Fig. S14 *A*–*C* and *G*–*I*). At 45 min after incubation at 32 °C, SBP-EGFP-E-cadherin was detectable on the cell surface or cell junction in the majority of cells (*SI Appendix*, Fig. S14 *D*–*F*, and *M*). Xyloside treatment did not cause defects in surface delivery of SBP-EGFP-E-cadherin after incubation at 32 °C (*SI Appendix*, Fig. S14 *J*–*L* and quantification in *SI Appendix*, Fig. S14*M*), suggesting that blocking PG synthesis does not block TGN-to-cell surface delivery of E-cadherin.

Finally, we performed a temperature shift experiment by incubating cells in the presence of biotin at 20 °C for 2 h and then 32 °C for 45 min. We found that the average number of punctate structures containing SBP-EGFP-Shh in each cell was significantly decreased after XYLT2 knockdown (Fig. 6 *K–Q*, and magnified views in Fig. 6 *N′–P′*). These analyses suggest that synthesis of PGs is important for the TGN export of the RUSH construct of Shh^FL^.

## Discussion

Regulating the release of newly synthesized signaling molecules by modulating their secretion can influence downstream signaling pathways in the target cells. Although fundamentally important, the underlying molecular mechanisms that mediate the biosynthetic trafficking of signaling molecules remain largely unclear. In this study, we analyzed the trafficking of a secreted signaling molecule, Shh. Based on the results from our study, we propose that the sorting and secretion of newly synthesized Shh is achieved in several steps (Fig. 5 *F–H*): 1) a cargo receptor, SURF4, interacts with the CW motif of Shh to package ShhN into COPII vesicles at the ER (step 1); 2) after being delivered to the Golgi, the SURF4-ShhN complex is dissociated (step 2); 3) the released SURF4 is retrieved to the ER by COPI vesicles (step 3); and 4) the released ShhN associates with PGs and is exported out of the TGN (step 4). We found that PGs compete with SURF4 to bind ShhN, and defects in PG synthesis enhance the association between SURF4 and ShhN at the Golgi. These analyses suggest that PGs are important factors regulating the displacement of SURF4 from ShhN at the Golgi (Fig. 5*F*). In addition to PGs, other factors such as other charged molecules may also contribute to the dissociation of SURF4 from ShhN at the Golgi (Fig. 5*H*).

Selective retention of proteins in the ER and capturing of cargo proteins in COPII vesicles have been shown to regulate the specificity of ER export (18). In addition to selective capture, cargo proteins can also exit the ER through bulk flow (18). In this mechanism, inclusion of cargo proteins into COPII vesicles occurs by default and is not dependent on receptors or export signals. Utilizing the RUSH assay, we found that EGFP without the CW motif is not transported from the ER to the Golgi after 30 min of biotin treatment (*SI Appendix*, Fig. S1 *N*–*P*). In contrast, the CW motif of Shh is sufficient for efficient export of EGFP out of the ER (Fig. 1 *A–I*). These analyses indicate that EGFP is not subject to significant forward transport by bulk flow.

After being delivered to the target compartment, cargo proteins need to be dissociated from their receptors, which are recycled back to the donor compartment to perform another round of cargo sorting. One mechanism that regulates this dissociation is a pH-sensitive ligand uncoupling mechanism (40, 41). This mechanism regulates the dissociation of soluble acid hydrolase precursor and their receptor Mannose 6-phosphate receptor (M6PR) in endosomes so that M6PR can be retrieved to the TGN to mediate the next cycle of hydrolase trafficking (41). In this study, we revealed a direct electrostatic interaction between the CW motif and negatively charged residues located in the predicted first luminal loop of SURF4. We provide evidence suggesting that PGs compete with SURF4 to interact with the CW motif in Shh at the Golgi, providing a way for dissociating cargo proteins from cargo receptors.

In addition to trafficking of Shh, SURF4 also mediates the export of other soluble proteins, including lipoproteins and PCSK9, from the ER. It also participates in ERES organization and interacts with amino-terminal hydrophobic-proline-hydrophobic motifs of soluble cargo proteins (19, 20, 22). We propose that the N-terminal tripeptide motif interacts with a domain on SURF4 that is distinct from the CW-motif binding site on SURF4. Another possibility is that the N-terminal tripeptide motif interact indirectly with SURF4 through an unknown cellular factor. As SURF4 contains a C-terminal retrieval signal (21), the C-terminal HA-tagged SURF4 may not has the maximal capacity as SURF4 without the HA tag. We found that SURF4-HA traffics together with ShhN from the ER to the Golgi and rescued the defects of ER-to-Golgi trafficking of ShhN in the RUSH assay. These analyses suggest that SURF4-HA is functional to promote ER-to-Golgi transport of ShhN, although it may not possess the maximum capacity as an untagged version of SURF4. KO of Shh causes embryonic lethality and induces defects in patterning of embryonic tissues, including the brain and eye, the spinal cord, the axial skeleton structures, and the limbs (43). KO of SURF4 also results in early embryonic lethality in mice with loss of all KO embryos between embryonic days 3.5 and 9.5 (44). Our results suggest that Shh is a key SURF4 client and that KO of SURF4 causes defects in the secretion of Shh, which contributes to defects in early embryonic development.

PGs are composed of core proteins linked to the GAG family of sugars, which includes heparan sulfate, dermatan sulfate, keratin sulfate, and chondroitin sulfate (45). All have been shown to interact with a variety of signaling molecules (45). These interactions regulate the free diffusion of signaling molecules and allow the PGs to function as signal coreceptors to regulate signal transduction (46). In *Drosophila*, a cell surface–located heparan sulfate proteoglycan, glypican, regulates the association of Hh with lipoproteins to facilitate the release of Hh in lipoprotein particles and thereby regulates the spread of Hh through a tissue (47). Heparan sulfate chains have been shown to regulate metalloprotease-mediated Shh release from producing cells (48) and Hh signaling in target cells (47). The CW motif of Shh is important for the interaction with heparan sulfate chains (37, 38). Mutations in this motif (R34A/K38A) in Shh reduce the affinity between Shh and proteoglycan in the cerebellum and decrease Shh-induced proliferation of granule cells in mice in situ (37). Interestingly, R34A/K38A mutations in Shh cause defects in proliferation of neural precursor cells but not in tissue patterning (39). In this study, we revealed that the CW motif can be sequentially recognized by SURF4 and PGs to mediate its surface delivery. In addition to ShhN, other CW motif–containing secretory proteins include bone morphogenetic protein (BMP) 8A, BMP8B, and Ihh. We hypothesize that the SURF4-proteoglycan relay mechanism may provide a general regulation for the ER-Golgi transport of CW motif–containing proteins.

How might PGs regulate TGN export of ShhN? We hypothesize that PGs regulate TGN export of ShhN by two possible nonmutually exclusive mechanisms: 1) deficiencies in PG synthesis induce delays in intra-Golgi transport of ShhN, which indirectly interfere with subsequent TGN export or 2) PG functions as a cargo receptor that regulates TGN sorting of Shh. The integral membrane proteoglycan Syndecan-1 (SDC1) acts as a cargo receptor that regulates TGN sorting of LPL (36). SDC1 and LPL are cosecreted in secretory vesicles enriched in sphingomyelin (SM) (36). It is proposed that physical features of the SDC1 transmembrane domain drives association with the SM-rich membrane of the TGN and that this association concentrates SDC1 and its associated LPL, thereby targeting SDC1 and bound LPL into the sphingomyelin secretion pathway (36). It would be interesting to test whether TGN sorting of ShhN is mediated by specific PGs and to analyze whether ShhN is also packaged into vesicles enriched with SM at the TGN.

The α-amino group of the cysteine residue at the N terminus of Shh is modified by palmitoylation catalyzed by Hh acyltransferase (15, 49). The palmitoylation modification requires an N-terminal cysteine with a free amino group (15). In the RUSH construct of Shh^FL^ or ShhN, the α-amino group of the cysteine residue at the N terminus of Shh forms a peptide bond with the SBP-GFP tag, suggesting the RUSH constructs of ShhN or Shh^FL^ utilized in our study are not modified by palmitoylation. The RUSH construct of ShhN can be efficiently secreted, indicating that the palmitoylation modification is not required for the secretion of Shh.

In this study, we used mammalian cells exogenously expressing a specific cargo protein as a system to investigate the molecular mechanisms of cargo sorting. This system is convenient to perform biochemical and cell biological approaches to reveal mechanistic insights. An important future direction is to investigate whether the identified sorting signals and cellular factors are physiologically important for Shh secretion in animals in vivo. Ligand production by tumor cells or the surrounding stroma has been demonstrated to activate the Hh signaling pathway to promote tumorigenesis (50–52). The protein interactions identified in our study that mediate the sorting and secretion of Shh provide therapeutic targets to down-regulate Hh signaling for cancer treatment by inhibiting the secretion of Shh.

## Materials and Methods

### Constructs, Reagents, Cell Culture, Transfection, and Immunofluorescence.

Cell lines, plasmids, siRNAs, antibodies, cell culture, transfection and immunofluorescence were described in the *SI Appendix*.

### RUSH Assay and Antibody Uptake Assay.

RUSH assays were performed as described previously (26). The antibody uptake assay was performed as described previously (53).

### Immunoprecipitation, Protein Purification, and Binding Assay.

Immunoprecipitation was performed as described (27). Purification of GST-tagged ShhN^25-49^ and GST-tagged SURF4^49-60^ was performed as described previously (54). GST pull down assays were performed as described previously (54). Peptide binding assay was performed as described previously (27).

### Sample Preparation for Label-Free Quantitative MS Analysis.

This procedure was performed as described previously (26).

### In Vitro Vesicle Formation Assay.

In vitro vesicular release assays were performed as described previously (27, 55).

## Supplementary Material

Supplementary File

Supplementary File

Supplementary File

Supplementary File

Supplementary File

## Data Availability

All study data are included in the article and/or supporting information.

## References

[1] P. W. Ingham, Y. Nakano, C. Seger, Mechanisms and functions of Hedgehog signalling across the metazoa. Nat. Rev. Genet. 12, 393–406 (2011). 21502959 10.1038/nrg2984

[2] R. Petrova, A. L. Joyner, Roles for Hedgehog signaling in adult organ homeostasis and repair. Development 141, 3445–3457 (2014). 25183867 10.1242/dev.083691 PMC4197719

[3] J. L. Mullor, P. Sánchez, A. Ruiz i Altaba, Pathways and consequences: Hedgehog signaling in human disease. Trends Cell Biol. 12, 562–569 (2002). 12495844 10.1016/s0962-8924(02)02405-4

[4] P. W. Ingham, M. Placzek, Orchestrating ontogenesis: variations on a theme by sonic hedgehog. Nat. Rev. Genet. 7, 841–850 (2006). 17047684 10.1038/nrg1969

[5] B. St-Jacques, M. Hammerschmidt, A. P. McMahon, Indian hedgehog signaling regulates proliferation and differentiation of chondrocytes and is essential for bone formation. Genes Dev. 13, 2072–2086 (1999). 10465785 10.1101/gad.13.16.2072 PMC316949

[6] M. J. Bitgood, L. Shen, A. P. McMahon, Sertoli cell signaling by Desert hedgehog regulates the male germline. Curr. Biol. 6, 298–304 (1996). 8805249 10.1016/s0960-9822(02)00480-3

[7] H. H. Yao, W. Whoriskey, B. Capel, Desert Hedgehog/Patched 1 signaling specifies fetal Leydig cell fate in testis organogenesis. Genes Dev. 16, 1433–1440 (2002). 12050120 10.1101/gad.981202 PMC186321

[8] M. Wijgerde, M. Ooms, J. W. Hoogerbrugge, J. A. Grootegoed, Hedgehog signaling in mouse ovary: Indian hedgehog and desert hedgehog from granulosa cells induce target gene expression in developing theca cells. Endocrinology 146, 3558–3566 (2005). 15878962 10.1210/en.2005-0311

[9] E. Dessaud, A. P. McMahon, J. Briscoe, Pattern formation in the vertebrate neural tube: a sonic hedgehog morphogen-regulated transcriptional network. Development 135, 2489–2503 (2008). 18621990 10.1242/dev.009324

[10] P. T. Yam, F. Charron, Signaling mechanisms of non-conventional axon guidance cues: The Shh, BMP and Wnt morphogens. Curr. Opin. Neurobiol. 23, 965–973 (2013). 24183376 10.1016/j.conb.2013.09.002

[11] A. P. McMahon, P. W. Ingham, C. J. Tabin, Developmental roles and clinical significance of hedgehog signaling. Curr. Top. Dev. Biol. 53, 1–114 (2003). 12509125 10.1016/s0070-2153(03)53002-2

[12] X. Chen , Processing and turnover of the Hedgehog protein in the endoplasmic reticulum. J. Cell Biol. 192, 825–838 (2011). 21357747 10.1083/jcb.201008090 PMC3051819

[13] I. A. Rose, J. V. Warms, An enzyme with ubiquitin carboxy-terminal esterase activity from reticulocytes. Biochemistry 22, 4234–4237 (1983). 6313036 10.1021/bi00287a012

[14] J. A. Porter, K. E. Young, P. A. Beachy, Cholesterol modification of hedgehog signaling proteins in animal development. Science 274, 255–259 (1996). 8824192 10.1126/science.274.5285.255

[15] J. A. Buglino, M. D. Resh, Hhat is a palmitoylacyltransferase with specificity for N-palmitoylation of Sonic Hedgehog. J. Biol. Chem. 283, 22076–22088 (2008). 18534984 10.1074/jbc.M803901200 PMC2494920

[16] J. Dancourt, C. Barlowe, Protein sorting receptors in the early secretory pathway. Annu. Rev. Biochem. 79, 777–802 (2010). 20533886 10.1146/annurev-biochem-061608-091319

[17] A. Schweizer, J. A. Fransen, T. Bächi, L. Ginsel, H. P. Hauri, Identification, by a monoclonal antibody, of a 53-kD protein associated with a tubulo-vesicular compartment at the cis-side of the Golgi apparatus. J. Cell Biol. 107, 1643–1653 (1988). 3182932 10.1083/jcb.107.5.1643 PMC2115344

[18] C. Barlowe, A. Helenius, Cargo capture and bulk flow in the early secretory pathway. Annu. Rev. Cell Dev. Biol. 32, 197–222 (2016). 27298089 10.1146/annurev-cellbio-111315-125016

[19] K. Saegusa, M. Sato, N. Morooka, T. Hara, K. Sato, SFT-4/Surf4 control ER export of soluble cargo proteins and participate in ER exit site organization. J. Cell Biol. 217, 2073–2085 (2018). 29643117 10.1083/jcb.201708115 PMC5987718

[20] B. T. Emmer , The cargo receptor SURF4 promotes the efficient cellular secretion of PCSK9. eLife 7, e38839 (2018). 30251625 10.7554/eLife.38839 PMC6156083

[21] X. Wang , Receptor-mediated ER export of lipoproteins controls lipid homeostasis in mice and humans. Cell Metab. 33, 350–366.e7 (2020). 33186557 10.1016/j.cmet.2020.10.020

[22] Y. Yin , Surf4 (Erv29p) binds amino-terminal tripeptide motifs of soluble cargo proteins with different affinities, enabling prioritization of their exit from the endoplasmic reticulum. PLoS Biol. 16, e2005140 (2018). 30086131 10.1371/journal.pbio.2005140 PMC6097701

[23] G. Boncompain , Synchronization of secretory protein traffic in populations of cells. Nat. Methods 9, 493–498 (2012). 22406856 10.1038/nmeth.1928

[24] A. D. Cardin, H. J. Weintraub, Molecular modeling of protein-glycosaminoglycan interactions. Arteriosclerosis 9, 21–32 (1989). 2463827 10.1161/01.atv.9.1.21

[25] M. Aridor, S. I. Bannykh, T. Rowe, W. E. Balch, Sequential coupling between COPII and COPI vesicle coats in endoplasmic reticulum to Golgi transport. J. Cell Biol. 131, 875–893 (1995). 7490291 10.1083/jcb.131.4.875 PMC2200014

[26] L. Niu , Atlastin-mediated membrane tethering is critical for cargo mobility and exit from the endoplasmic reticulum. Proc. Natl. Acad. Sci. U.S.A. 116, 14029–14038 (2019). 31239341 10.1073/pnas.1908409116 PMC6628656

[27] X. Tang , Molecular mechanisms that regulate export of the planar cell-polarity protein Frizzled-6 out of the endoplasmic reticulum. J. Biol. Chem. 295, 8972–8987 (2020). 32376691 10.1074/jbc.RA120.012835 PMC7335806

[28] Y. Huang , An in vitro vesicle formation assay reveals cargo clients and factors that mediate vesicular trafficking. Proc. Natl. Acad. Sci. U.S.A. 118, e2101287118 (2021). 34433667 10.1073/pnas.2101287118 PMC8536394

[29] Y. Guo, A. D. Linstedt, COPII-Golgi protein interactions regulate COPII coat assembly and Golgi size. J. Cell Biol. 174, 53–63 (2006). 16818719 10.1083/jcb.200604058 PMC2064162

[30] F. Yang, T. Li, Z. Peng, Y. Liu, Y. Guo, The amphipathic helices of Arfrp1 and Arl14 are sufficient to determine subcellular localizations. J. Biol. Chem. 295, 16643–16654 (2020). 32972971 10.1074/jbc.RA120.014999 PMC7864062

[31] S. Otte, C. Barlowe, Sorting signals can direct receptor-mediated export of soluble proteins into COPII vesicles. Nat. Cell Biol. 6, 1189–1194 (2004). 15516922 10.1038/ncb1195

[32] W. J. Belden, C. Barlowe, Role of Erv29p in collecting soluble secretory proteins into ER-derived transport vesicles. Science 294, 1528–1531 (2001). 11711675 10.1126/science.1065224

[33] M. Varadi , AlphaFold protein structure database: Massively expanding the structural coverage of protein-sequence space with high-accuracy models. Nucleic Acids Res. 50, D439–D444 (2021). 10.1093/nar/gkab1061 PMC8728224 34791371

[34] J. Jumper , Highly accurate protein structure prediction with AlphaFold. Nature 596, 583–589 (2021). 34265844 10.1038/s41586-021-03819-2 PMC8371605

[35] H. Hughes , Organisation of human ER-exit sites: Requirements for the localisation of Sec16 to transitional ER. J. Cell Sci. 122, 2924–2934 (2009). 19638414 10.1242/jcs.044032 PMC2724609

[36] E. L. Sundberg, Y. Deng, C. G. Burd, Syndecan-1 mediates sorting of soluble lipoprotein lipase with Sphingomyelin-rich membrane in the Golgi apparatus. Dev. Cell 51, 387–398.e4 (2019). 31543446 10.1016/j.devcel.2019.08.014 PMC6832887

[37] J. B. Rubin, Y. Choi, R. A. Segal, Cerebellar proteoglycans regulate sonic hedgehog responses during development. Development 129, 2223–2232 (2002). 11959830 10.1242/dev.129.9.2223

[38] P. Farshi , Dual roles of the Cardin-Weintraub motif in multimeric Sonic hedgehog. J. Biol. Chem. 286, 23608–23619 (2011). 21572042 10.1074/jbc.M110.206474 PMC3123124

[39] J. A. Chan , Proteoglycan interactions with Sonic Hedgehog specify mitogenic responses. Nat. Neurosci. 12, 409–417 (2009). 19287388 10.1038/nn.2287 PMC2676236

[40] C. G. Davis , Acid-dependent ligand dissociation and recycling of LDL receptor mediated by growth factor homology region. Nature 326, 760–765 (1987). 3494949 10.1038/326760a0

[41] P. Ghosh, N. M. Dahms, S. Kornfeld, Mannose 6-phosphate receptors: New twists in the tale. Nat. Rev. Mol. Cell Biol. 4, 202–212 (2003). 12612639 10.1038/nrm1050

[42] P. Paroutis, N. Touret, S. Grinstein, The pH of the secretory pathway: Measurement, determinants, and regulation. Physiology (Bethesda) 19, 207–215 (2004). 15304635 10.1152/physiol.00005.2004

[43] C. Chiang , Cyclopia and defective axial patterning in mice lacking Sonic hedgehog gene function. Nature 383, 407–413 (1996). 8837770 10.1038/383407a0

[44] B. T. Emmer , Murine Surf4 is essential for early embryonic development. PLoS One 15, e0227450 (2020). 31978056 10.1371/journal.pone.0227450 PMC6980569

[45] T. Annaval , Heparan sulfate proteoglycans biosynthesis and post synthesis mechanisms combine few enzymes and few core proteins to generate extensive structural and functional diversity. Molecules 25, 4215 (2020). 10.3390/molecules25184215 PMC7570499 32937952

[46] P. Lu, V. M. Weaver, Z. Werb, The extracellular matrix: A dynamic niche in cancer progression. J. Cell Biol. 196, 395–406 (2012). 22351925 10.1083/jcb.201102147 PMC3283993

[47] J. Briscoe, P. P. Thérond, The mechanisms of Hedgehog signalling and its roles in development and disease. Nat. Rev. Mol. Cell Biol. 14, 416–429 (2013). 23719536 10.1038/nrm3598

[48] T. Dierker, R. Dreier, A. Petersen, C. Bordych, K. Grobe, Heparan sulfate-modulated, metalloprotease-mediated sonic hedgehog release from producing cells. J. Biol. Chem. 284, 8013–8022 (2009). 19176481 10.1074/jbc.M806838200 PMC2658095

[49] R. B. Pepinsky , Identification of a palmitic acid-modified form of human Sonic hedgehog. J. Biol. Chem. 273, 14037–14045 (1998). 9593755 10.1074/jbc.273.22.14037

[50] D. M. Berman , Widespread requirement for Hedgehog ligand stimulation in growth of digestive tract tumours. Nature 425, 846–851 (2003). 14520411 10.1038/nature01972

[51] R. L. Yauch , A paracrine requirement for hedgehog signalling in cancer. Nature 455, 406–410 (2008). 18754008 10.1038/nature07275

[52] M. Niyaz, M. S. Khan, S. Mudassar, Hedgehog signaling: An Achilles’ heel in cancer. Transl. Oncol. 12, 1334–1344 (2019). 31352196 10.1016/j.tranon.2019.07.004 PMC6664200

[53] R. Natarajan, A. D. Linstedt, A cycling cis-Golgi protein mediates endosome-to-Golgi traffic. Mol. Biol. Cell 15, 4798–4806 (2004). 15331763 10.1091/mbc.E04-05-0366 PMC524728

[54] Y. Guo, G. Zanetti, R. Schekman, A novel GTP-binding protein-adaptor protein complex responsible for export of Vangl2 from the trans Golgi network. eLife 2, e00160 (2013). 23326640 10.7554/eLife.00160 PMC3539332

[55] X. Tang, F. Yang, Y. Guo, Cell-free reconstitution of the packaging of cargo proteins into vesicles at the *trans* Golgi network. Bio Protoc. 10, e3537 (2020). 10.21769/BioProtoc.3537 PMC7842834 33659511

