# Supplementary material for: A SURF4-to-proteoglycan relay mechanism that mediates the sorting and secretion of a tagged variant of sonic hedgehog
Source: Proc Natl Acad Sci U S A. 2022 Mar 10;119(11):e2113991119. doi: 10.1073/pnas.2113991119 (PMC8931250; doi:10.1073/pnas.2113991119)
Supplement: Supplementary File [file pnas.2113991119.sapp.pdf]

Supplemental Information (SI) Appendix

A SURF4-to-proteoglycan relay mechanism that mediates the sorting and secretion of a tagged-variant of sonic hedgehog

Xiao Tang[1], Rong Chen[2], Vince St Dollente Mesias[3], Tingxuan Wang[1], Ying Wang[4], Kristina Poljak[5], Xinyu Fan[1], Hanchi Miao[6], Junjie Hu[6], Liang Zhang[4], Jinqing Huang[3], Shuhuai Yao[2,7], Elizabeth A. Miller[5], and Yusong Guo[1,8,9,*]

1. Division of Life Science and State Key Laboratory of Molecular Neuroscience, The Hong Kong University of Science and Technology, Hong Kong, China
2. Department of Chemical and Biological Engineering, The Hong Kong University of Science and Technology, Hong Kong, China
3. Department of Chemistry, The Hong Kong University of Science and Technology, Clear Water Bay, Hong Kong, China.
4. Department of Biomedical Sciences, The City University of Hong Kong, Hong Kong, China
5. Cell Biology Division, Medical Research Council Laboratory of Molecular Biology, Cambridge CB2 0QH, UK
6. National Laboratory of Biomacromolecules, Chinese Academy of Sciences Center for Excellence in Biomacromolecules, Institute of Biophysics, Chinese Academy of Sciences, Beijing 100101, China
7. Department of Mechanical and Aerospace Engineering, The Hong Kong University of Science and Technology, Hong Kong, China
8. Shenzhen Research Institute, Hong Kong University of Science and Technology, Shenzhen 518057, China
9. Southern Marine Science and Engineering Guangdong Laboratory (Guangzhou), Guangzhou 511458, China

* Correspondence: Yusong Guo, guoyusong@ust.hk

Includes:

SI Materials and Methods

SI Figures, Figure S1-S14

SI Materials and Methods

Constructs, reagents, cell culture, transfection and immunofluorescence

HeLa cells and HEK293T cell lines were kindly provided by the University of California-Berkeley Cell Culture Facility and were confirmed by short tandem repeat profiling. All cell lines were tested negative for Mycoplasma contamination. HeLa and HEK293T cells were cultured in Dulbecco's Modified Eagle Medium (DMEM) containing 10% fetal bovine serum and 1% penicillin streptomycin mix (Invitrogen). HEK 293Trex and HEK 293Trex SURF4 KO cell lines were maintained in Gibco DMEM containing 5 μg/ml blasticidin and 10% FBS.

The cDNA encoding mouse ShhN, human SURF4, human IGF2 and human XYLT2 were ordered from BGI (Beijing, China). The plasmids encoding C-terminal 3xHA-tagged $ShhN^{1-198}$, C-terminal GST-tagged Shh^{25-49}, C-terminal 3xHA-tagged IGF2, C-terminal 3xHA-tagged SURF4, C-terminal 3xMyc-tagged SURF4, N-terminal GST-tagged $SURF4^{49-60}$, N-terminal 3xMyc-tagged XYLT2, Str-KDEL_SBP-EGFP-$ShhN^{25-198}$, Str-KDEL_SBP-EGFP-Shh^{FL}, Str-KDEL_SBP-EGFP-E-cadherin and truncated versions of ShhN were generated by standard molecular cloning procedures. The N-terminus of SBP-EGFP tag is followed by a signal sequence derived from IL-2 (1). The plasmids encoding mutated versions of ShhN and SURF4 were generated by QuickChange II site-directed mutagenesis using plasmids encoding ShhN-HA, Str-KDEL_SBP-EGFP-ShhN and SURF4-3xMyc as templates. The plasmids encoding siRNA-resistant SURF4-HA were generated by QuickChange II site-directed mutagenesis using plasmid encoding SURF4-HA as template.

siRNAs against SURF4 and XYLT2 were purchased from Ribo-bio (Guangzhou, China). The target sequence of the two siRNAs against SURF4 is GCAGGAACTTCGTGCAGTA and GCATCCGTATGTGGTTCCA respectively. The target sequence of the two siRNAs against XYLT2 is CTGGTAGTGTGGAGCTTCA and GCGTGCACCTGTATTTCTA respectively. The commercial antibodies were rabbit anti-HA (Cell Signaling, catalogue number 3724), mouse anti-HA (Biolegend, catalogue number 901501), mouse anti-Myc (Cell Signaling, catalogue number 2276), mouse anti-PDI (Enzo, catalogue number ADI-SPA-891-F), sheep anti-TGN46 (BIO-RAD, catalogue number AHP500G), rabbit anti-ERGIC53 for the immunofluorescence analysis (Sigma-Aldrich, catalogue number E1031) and mouse anti-GM130 (BD Bioscience, catalogue number 610823). Rabbit anti-SEC22B antibodies and rabbit anti-ERGIC53 antibodies for the immunoblot analyses were kindly provided by Prof. Randy Schekman (University of California, Berkeley, CA, USA). Rabbit anti-SURF4 antibodies were kindly provided by Prof. Xiaowei Chen (Peking University, China). Rabbit anti-GFP antibodies were kindly provided by Prof. Robert Qi (Hong Kong University of Science and Technology, Hong Kong, SAR).

Transfection of siRNA or DNA constructs into HeLa cells, or HEK293T cells and immunofluorescence were performed as described previously (2). Images were acquired with a Zeiss Axio Observer Z1 microscope system (Carl Zeiss, Germany) equipped with an ORCA Flash 4.0 camera (Hamamatsu, Japan)

or Leica STED TCS SP5 II Confocal Laser Scanning Microscope (Leica, Germany). To quantify the colocalization between ERGIC53 and GM130, the juxta-nuclear area labelled by GM130 was outlined manually using the freehand selections function in Fiji as the region of interest (ROI). To quantify the colocalization between SURF4-HA and GM130 or ERGIC53, the juxta-nuclear area labelled by SBP-EGFP-ShhN was outlined manually in Fiji as the ROI. To quantify the colocalization between ShhN and GM130, the juxta-nuclear area labelled by ShhN was outlined manually in Fiji as the ROI. All of the pixels in the ROI were not saturated. The Pearson's R value (no threshold) in the ROI between two channels was then calculated using Coloc 2 in Fiji.

For CRISPR experiments, single-guide RNA (sgRNA) sequences ligated into pX458 (pSpCas9 BB-2A-GFP) plasmids were purchased from GenScript. Transfections were performed with TransitIT-293 (Mirus Bio) per manufacturer's instructions. Clonal cell lines were derived by diluting cell suspensions to a single cell per well and expanding individual wells. Genotyping of clonal cell lines was performed by Sanger sequencing of target site PCR amplicons of genomic DNA isolated by Puregene kit (Quiagene). sgRNAs were as follows: SURF4, 5'AGTCGCGCTGCTCGCTCCAC3' targeting exon 1.

Retention Using Selective Hook (RUSH) assay, antibody uptake assay and permeabilized cell assay

RUSH assays were performed by treating HeLa cells transfected with plasmids encoding Str-KDEL and different versions of SBP-EGFP-ShhN in complete medium containing 40 μM biotin (Sigma-Aldrich) and 100 ng/μl cycloheximide (Sigma-Aldrich) for the indicated time. Cells were then fixed by 4% PFA mounted on glass slides by ProLong™ Gold Antifade Mountant with DAPI (Invitrogen) for microscope analysis.

To analyse the secretion of ShhN, HEK293T cells transfected with plasmids encoding Str-KDEL and different versions of SBP-EGFP-ShhN were treated by 100 ng/μl cycloheximide and 40 μM biotin in medium without FBS addition for the indicated time. Then the secreted proteins were collected by TCA precipitation. The cells were collected and lysed by HKT buffer (100 mM KCl, 20 mM Hepes, pH 7.2, 0.5% Triton X-100). The bound proteins and cell lysates were analysed by immunoblotting.

For antibody uptake assays, HeLa cells were treated (or not) with 2.5 mM xyloside in complete medium. 24hr after xyloside treatment, cells were transfected with plasmids encoding Str-KDEL_SBP-EGFP-HA-Shh^{FL}. On day 2 after xyloside treatment, cells were incubated without biotin or with 40 μM biotin and 100 ng/μl cycloheximide in complete medium for 1 hr. After incubation, mouse anti-HA antibodies were added to the incubation medium at a 1:200 dilution to label the ShhN fusion construct that had been delivered to the cell surface. After an additional incubation for 40min at 37 °C, cells were fixed for 15 min with 4% paraformaldehyde in PBS and then a standard immunofluorescence procedure was performed using rabbit anti-GFP antibodies as the primary antibodies.

Permeabilized cell assay was performed as described previously (3). Briefly, HeLa cells transfected with Str-KDEL_SBP-EGFP-ShhN were treated with 0.04 mM biotin at 37 °C for 4 min, followed by three washes

in cold KOAc buffer (110 mM KOAc, 2 mM $Mg(OAc)_2$, 20 mM Hepes, pH 7.2). Then cells were permeabilized by 0.03 mg/ml digitonin in KOAc buffer for 6 min at room temperature. The permeabilized cells were washed with cold KOAc buffer. After 5 min of incubation on ice with cold 0.5 M KOAc buffer followed by three washes in cold KOAc buffer to remove cytosolic proteins, the permeabilized cells were then incubated at 37 °C for 15 min in KOAc buffer containing 2 mg/ml rat liver cytosol, 0.04 mM biotin, 500 μM GDP/GTPγS, and an ATP regeneration system (40 mM creatine phosphate, 0.2 mg/ml of creatine phosphokinase, and 1 mM ATP). The cells were then washed with cold KOAc buffer, fixed, and stained with specific antibodies.

Immunoprecipitation, protein purification, and binding assay

Immunoprecipitation of HA-tagged ShhN was performed by incubating 200 μl of 0.5 mg/ml cell lysates from HEK293T cells transfected with ShhN-HA in HKT buffer with 10 μl of compact anti-HA agarose affinity beads with mixing at 4 °C overnight. After incubation, the beads were washed 4 times with 1 ml of HK buffer (100 mM KCl, 20 mM Hepes, pH 7.2), and the bound material was analyzed by Coomassie blue staining and immunoblotting.

Binding assays between Myc-tagged SURF4 or Myc-tagged $SURF4^{ED\text{-}AA}$ and HA-tagged ShhN or $ShhN^{\Delta 33\text{-}39}$ were performed by treating HEK293T cells co-transfected with plasmids encoding the indicated proteins in 1 × PBS containing 2 mM dithiobis[succinimidylpropionate] (DSP) and 2 mM $CaCl_2$ at room temperature for 30 min, and then quenched with 25 mM Tris-HCl, pH 7.5. 200 μl of 0.5 mg/ml cell lysates were incubated with 10 μl of compact anti-HA agarose affinity beads with mixing at 4 °C overnight. After incubation, the beads were washed 4 times with 1 ml of HK buffer (100 mM KCl, 20 mM Hepes, pH 7.2), and the bound material was analyzed by immunoblotting.

Purification of GST-tagged $ShhN^{25\text{-}49}$ and GST-tagged $SURF4^{49\text{-}60}$ was performed as described previously (4). GST pull-down assays were carried out with 10 μl of compact GSH beads bearing around 5 μg of GST-tagged $ShhN^{25\text{-}49}$. The beads were incubated with 200 μl of 0.5 mg/ml of cell lysates from HEK293T cells transfected with HA-SURF4 in HKT buffer at pH 6.0 or 7.2 with mixing at 4 °C overnight. After incubation, the beads were washed three times with 500 μl of HKT buffer and twice with 500 μl of HK buffer, and the bound material was analyzed by immunoblotting.

Peptide binding assay was performed as described previously (2). Synthetic CW peptides (KRRHPKKC), CW^{MT} peptides (AAAHPAAC), $SURF4^{49\text{-}61}$ peptides (SEQRDYIDTTWNC), or RRFR peptides (VRRFRYPERPC) were purchased from GenScript and coupled to thiopyridone-Sepharose 6B beads (Sigma-Aldrich) via the added C-terminal cysteine residue. For binding experiments, 2 μg purified GST-tagged $SURF4^{49\text{-}60}$ or $Shh^{25\text{-}49}$-GST was preincubated at 4 °C for 30 min in a total volume of 15 ml HK buffer. After incubation, 15 ml buffer containing around 5 μl beads containing 5 nmol of peptides was added to the

reaction mixture for 1 h at 4 °C. The beads were washed four times with 500 μl of HK buffer and analyzed by immunoblotting.

Label-free quantitative mass spectrometry

Mass spectrometry was performed to identify the proteins involved in Shh secretion. After transfecting plasmids encoding Shh-HA or IGF2-HA into HEK293T cells, the immunoprecipitation was processed, and the bound proteins were analyzed by Coomassie Blue (Bio-Safe^{TM} Coomassie-G250) staining. The protein gel was cut into small fragments and washed with 25 mM NH_4HCO_3/50% acetonitrile at room temperature for 15 min for three times. Then the gel fragments were shrunken by acetonitrile at room temperature for 15 min and dried by speed vacuum. The dried protein gel pieces were reduced by 0.1 M NH_4HCO_3 containing 10 mM TCEP at 55 °C for 45 min and alkylated by 0.1 M NH_4HCO_3 containing 55 mM Indoacetamide at room temperature in the dark for 45 min. After that, the gel pieces were washed by 0.1 M NH_4HCO_3 and repeated the steps of shrink and dry. Then the proteins were digested by 50 mM NH_4HCO_3 containing 20 ng/μl sequencing grade modified trypsin (Promega, number V511A) on ice for 45 min and incubated in 50 mM NH_4HCO_3 at 37 °C overnight. 25 mM NH_4HCO_3 and 60% acetonitrile containing 5% formic acid were used to exact the peptides respectively. Then the samples were dried with speed vacuum. The dried peptides were dissolved into 0.1% trifluoroacetic acid to remove the surfactant, desalted using pierce C18 spin column and dried by speed vacuum. Finally, the resulted peptides were analyzed by Mass Spectrometer. The tandem mass spectra were then subject to protein identification and label-free quantification by Proteome Discoverer. The proteins that were associated with the Shh or IGF2 were identified by comparing the peak intensity of the identified protein in the experimental group with the control group.

***In vitro* vesicle formation assay**

In vitro vesicular release assays were performed as described previously (2, 5). Briefly, Day 1 after transfection with plasmids encoding HA-tagged different version of ShhN, HEK293T cells grown in one 10-cm dish at around 90% confluence were permeabilized in 3 ml of ice-cold KOAc buffer containing 40 μg/ml digitonin on ice for 5 min, and the semi-intact cells were then sedimented by centrifugation at 300 × g for 3 min at 4 °C. The cell pellets were washed twice with 1 ml of KOAc buffer and resuspended in 100 μl of KOAc buffer. The budding assay was performed by incubating semi-intact cells (around 0.02 OD/reaction) with 2 mg/ml of rat liver cytosol in a 100 μl reaction mixture containing 200 μM GTP and an ATP regeneration system in the presence or absence of 0.5 μg of Sar1A (H79G). After incubation at 32 °C for 1 h, the reaction mixture was centrifuged at 14,000 × g to remove cell debris and large membranes. The medium-speed supernatant was then centrifuged at 100,000 × g to sediment small vesicles. The pellet fraction was then analyzed by immunoblotting. For density gradient flotation assays, the pellet fraction was resuspended in 100 μl of 35% OptiPrep and overlaid with 700 μl of 30% OptiPrep and 30 μl of KOAc buffer.

The samples were centrifuged at 55,000 rpm in a TLS55 rotor in a Beckman ultracentrifuge for 2 hr at 4 °C. After centrifugation, fractions were collected from the top to the bottom of the tube, and the top fraction was analyzed by SDS-PAGE and immunoblotting.

Isothermal titration calorimetry (ITC)

We utilized a commercial isothermal titration calorimeter (PEAQ-ITC system, MicroCal, Malvern Panalytical, United Kingdom) to perform titration experiments. In these experiments, a 500 μM solution of CW peptide in a diluted binding buffer (10 mM PBS, 0.5 mM HEPES, 6.3 mM sorbitol, 1.75 mM KOAc, 25 μM $Mg(OAc)_2$, 25 μM BSA, 0.003% Triton, pH 7.2) was loaded into the syringe of the ITC instrument and a 50 μM solution of GST-SURF4 (49-60) in the same buffer was in the calorimetric cell. Similarly, GST was titrated with the CW peptide. During each ITC experiment, 19 injections of CW peptide into the calorimetric cell were carried out while the duration of each injection was 4 s and the time between the injections was 150 s. The volume of each injection was 2 μL and the stirring speed was maintained at 750 rpm. Control experiments such as peptide to buffer and buffer to protein titrations were also done to account for non-specific interactions and heat of dilution. The binding constant (K_d) was derived by fitting the data with one set of site model using the MicroCal PEAQ-ITC analysis software.

SI References

1. G. Boncompain *et al.*, Synchronization of secretory protein traffic in populations of cells. *Nat Methods* **9**, 493-498 (2012).
2. X. Tang *et al.*, Molecular mechanisms that regulate export of the planar cell-polarity protein Frizzled-6 out of the endoplasmic reticulum. *J Biol Chem* 10.1074/jbc.RA120.012835 (2020).
3. F. Yang, T. Li, Z. Peng, Y. Liu, Y. Guo, The amphipathic helices of Arfrp1 and Arl14 are sufficient to determine subcellular localizations. *J Biol Chem* **295**, 16643-16654 (2020).
4. Y. Guo, G. Zanetti, R. Schekman, A novel GTP-binding protein-adaptor protein complex responsible for export of Vangl2 from the trans Golgi network. *Elife* **2**, e00160 (2013).
5. X. Tang, F. Yang, Y. Guo, Cell-free Reconstitution of the Packaging of Cargo Proteins into Vesicles at the trans Golgi Network. *Bio Protoc* **10**, e3537 (2020).

Figure S1

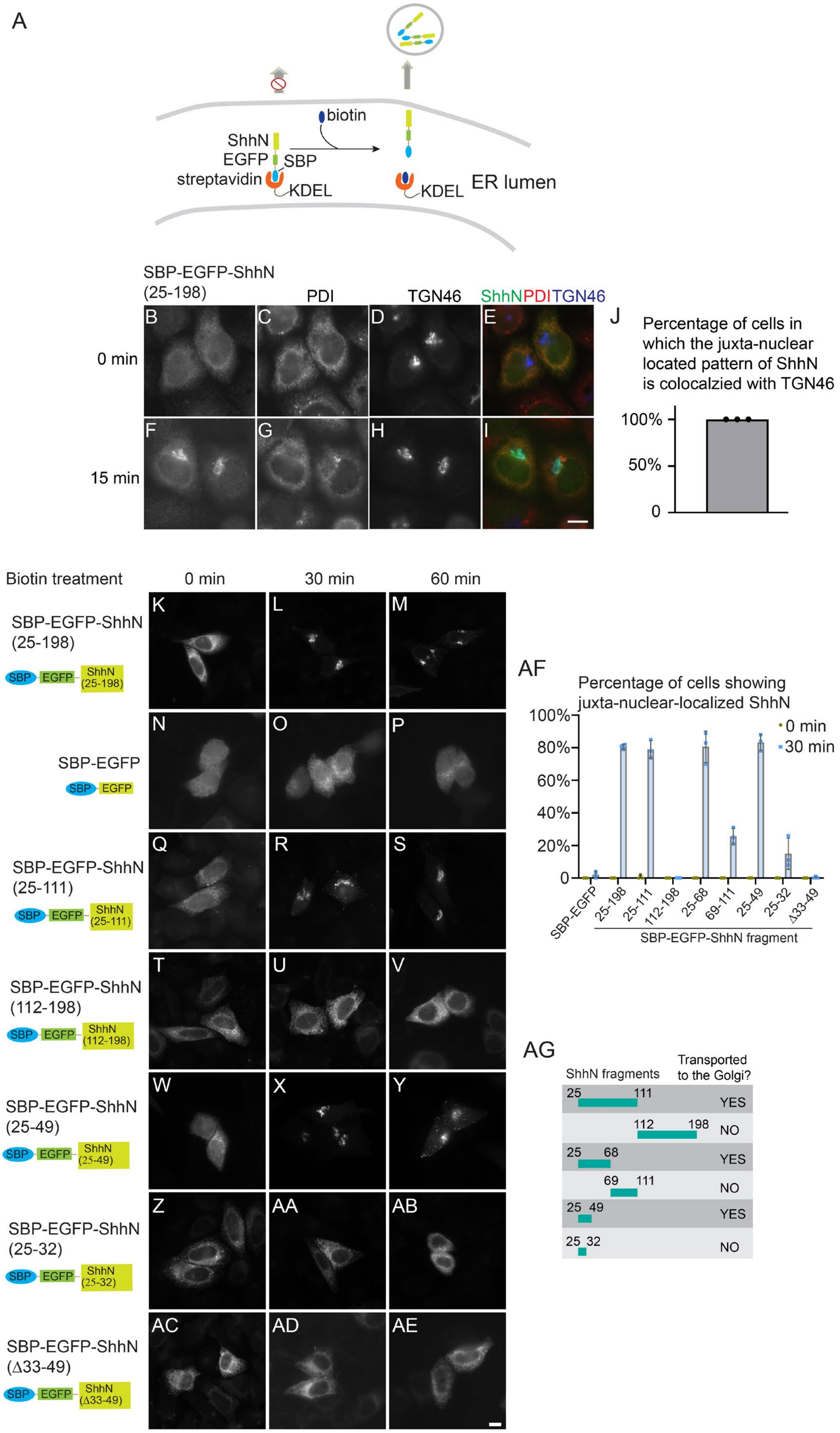
A
biotin
ShhN
EGFP
SBP
streptavidin
KDEL
KDEL
ER lumen
SBP-EGFP-ShhN (25-198)
PDI
TGN46
ShhN PDI TGN46
B C D E
0 min
F G H I
15 min
J
Percentage of cells in which the juxta-nuclear located pattern of ShhN is colocalzied with TGN46
100%
50%
0
Biotin treatment
0 min
30 min
60 min
SBP-EGFP-ShhN (25-198)
SBP-EGFP
SBP-EGFP-ShhN (25-111)
SBP-EGFP-ShhN (112-198)
SBP-EGFP-ShhN (25-49)
SBP-EGFP-ShhN (25-32)
SBP-EGFP-ShhN (Δ33-49)
K L M N O P Q R S T U V W X Y Z AA AB AC AD AE
AF
Percentage of cells showing juxta-nuclear-localized ShhN
0 min
30 min
100% 80% 60% 40% 20% 0
SBP-EGFP 25-198 25-111 112-198 25-68 69-111 25-49 25-32 Δ33-49
SBP-EGFP-ShhN fragment
AG
ShhN fragments
Transported to the Golgi?
25 111 YES
112 198 NO
25 68 YES
69 111 NO
25 49 YES
25 32 NO

Figure S1. Residues 33-49 in ShhN are important for ER export of ShhN. A. Diagram demonstrating the RUSH assay. **B-I.** HeLa cells were transfected with plasmids encoding SBP-EGFP-ShhN$^{25\text{-}198}$ and Streptavidin-KDEL. Day 1 after transfection, cells were left untreated or treated with biotin for 15 min. The localizations of the indicated proteins were then analyzed by immunofluorescence. *Scale bar,* 10µm. *Magnification,* 63x. **J.** Quantification showing that ShhN colocalized with TGN46 among all of the cells showing juxta-nuclear located pattern of SBP-EGFP-ShhN 15 min after biotin treatment (n=3). **K-AE.** HeLa cells were transfected with plasmids encoding Str-KDEL and SBP-EGFP-ShhN$^{25\text{-}198}$ (K-M) or SBP-EGFP (N-P) or SBP-EGFP tagged different truncated versions of ShhN (Q-AE). Day 1 after transfection, the localization of the different versions of RUSH constructs containing ShhN was analysed after incubation with biotin for the indicated time. *Scale bar,* 10 µm. *Magnification,* 63x. **AF.** Quantification of the percentage of cells showing juxta-nuclear-accumulated EGFP signal 30 min after biotin treatment (mean ± S.D.; n = 3; >100 cells counted for each time point). **AG.** Diagram depicting the fragment of ShhN that can be transported to the Golgi.

Figure S2

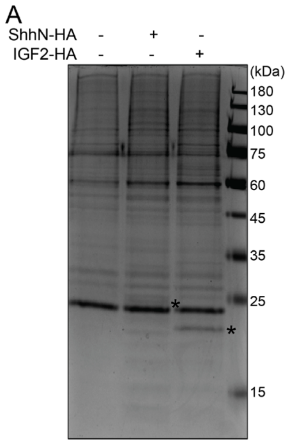

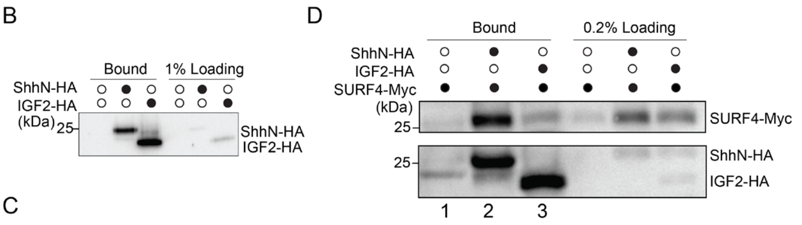

C

Gene name	Number of Unique Peptides	The Control Group	The IGF2 Group	The ShhN Group	Abundance Ratio ShhN/IGF2
BHMT	1	NA	NA	2063.184814	NA
GRIA1	1	NA	NA	19788.97656	NA
PPIB	1	NA	NA	8944.745117	NA
SURF4	1	NA	NA	7037.842773	NA
VPS51	1	NA	NA	45412.09375	NA
RPS15A	2	156239.4744	61158.17973	293199.9219	4.794124403
CLINT1	2	247624.8556	78529.78931	274752.8125	3.498708133
SHH	2	488036.6143	1262229.987	4295499.25	3.403103471

Figure S2. SURF4 was identified to be a binding partner of ShhN. A-B. HEK293T cells were left untransfected or transfected with plasmids encoding ShhN$^{1\text{-}198}$-HA (ShhN-HA) or IGF2-HA. Day 1 after transfection, cells were lysed and ShhN-HA or IGF2-HA were immunoprecipitated and analysed by SDS-PAGE and coomassie blue staining (A) or immunoblotting with anti-HA antibodies (B). Asterisks indicate the position of IGF2-HA or ShhN-HA. **C.** A label-free quantification was performed to quantify the intensity of unique peptides to measure the abundance of proteins identified in the mass spectrometry. A table showing the list of proteins in the eluted fraction whose abundance in the ShhN-HA group was calculated to be more than 3-fold that detected in the IGF2-HA group. **D.** HEK293T cells were transfected with plasmids encoding the constructs indicated. Day 1 after transfection, cells were treated with 2 mM DSP, and cell lysates were incubated with beads conjugated with anti-HA antibodies. The bound proteins indicated were analyzed by immunoblotting with anti-HA or anti-Myc antibodies.

Figure S3

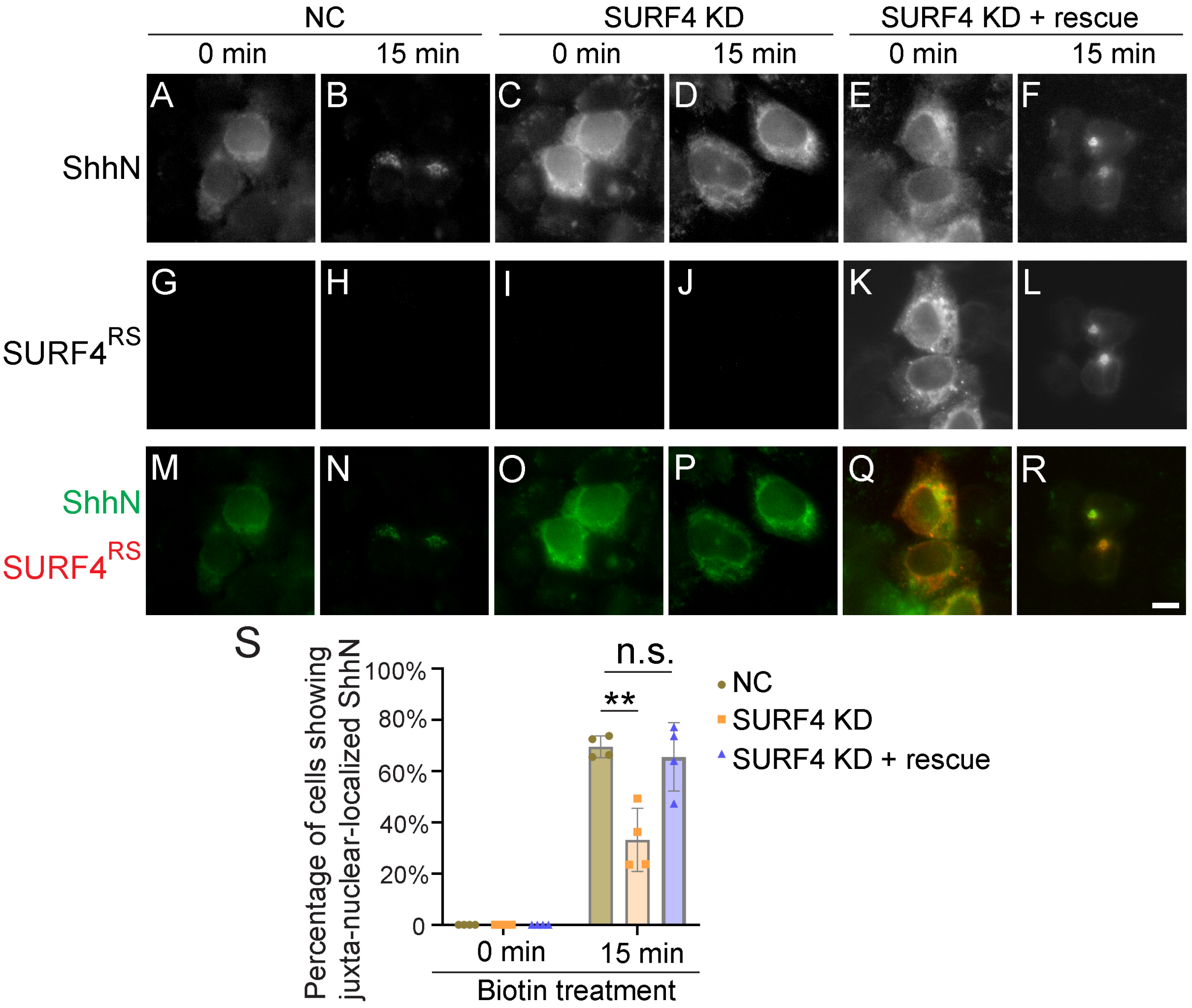

Figure S3. Expressing the siRNA resistant SURF4 recues the defects of ER-to-Golgi transport of ShhN in SURF4 knockdown cells.

A-R. HeLa cells were transfected with negative control (NC) siRNA (A-B, G-H and M-N) or siRNAs against SURF4 (C-F, I-L and O-R). 48hr after transfection, cells were re-transfected with plasmids encoding Str-KDEL_ SBP-EGFP-ShhN$^{25\text{-}198}$ (A-D, G-J and M-P), or siRNA-resistant SURF4^{RS}-HA and Str-KDEL_ SBP-EGFP-ShhN$^{25\text{-}198}$ (E-F, K-L and Q-R). On day 3 after knockdown, cells were incubated with biotin for the indicated time and the localization of ShhN was analyzed. *Scale bar,* 10 µm. *Magnification,* 63x. **S.** Quantification of the percentage of cells showing juxta-nuclear-localized SBP-EGFP-ShhN$^{25\text{-}198}$ ($n = 4$, mean ± S.D., over 100 cells were quantified in each experimental group). **, $p < 0.01$; n.s., not significant.

Figure S4

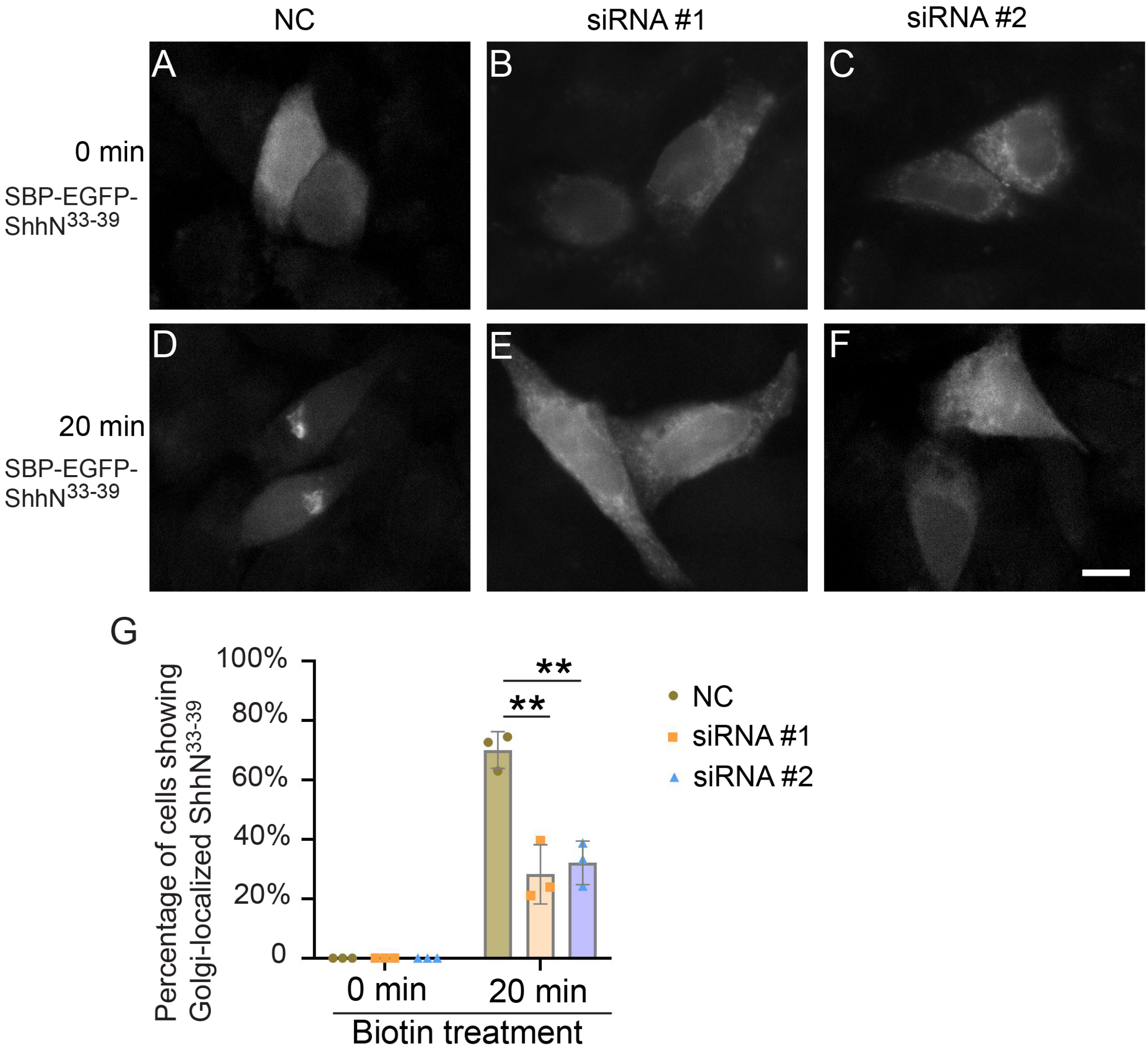

Figure S4. SURF4 regulates ER-to-Golgi trafficking of SBP-EGFP-ShhN$^{33\text{-}39}$. A-F. HeLa cells were transfected with negative control (NC) siRNA (A, D) or two different siRNAs against SURF4 (B, E, C, F). 24hr after transfection, cells were re-transfected with plasmids encoding SBP-EGFP-ShhN$^{33\text{-}39}$ and Str-KDEL. On day 3 after knockdown, cells were incubated with biotin for the indicated time and the localization of the indicated proteins was analysed. *Scale bar,* 10 μm. *Magnification,* 63x. **G.** Quantifications of the percentage of cells showing juxta-nuclear-localized SBP-EGFP-ShhN$^{33\text{-}39}$ (mean ± S.D.; n = 3; >100 cells counted for each experiment). **, $p < 0.01$.

Figure S5

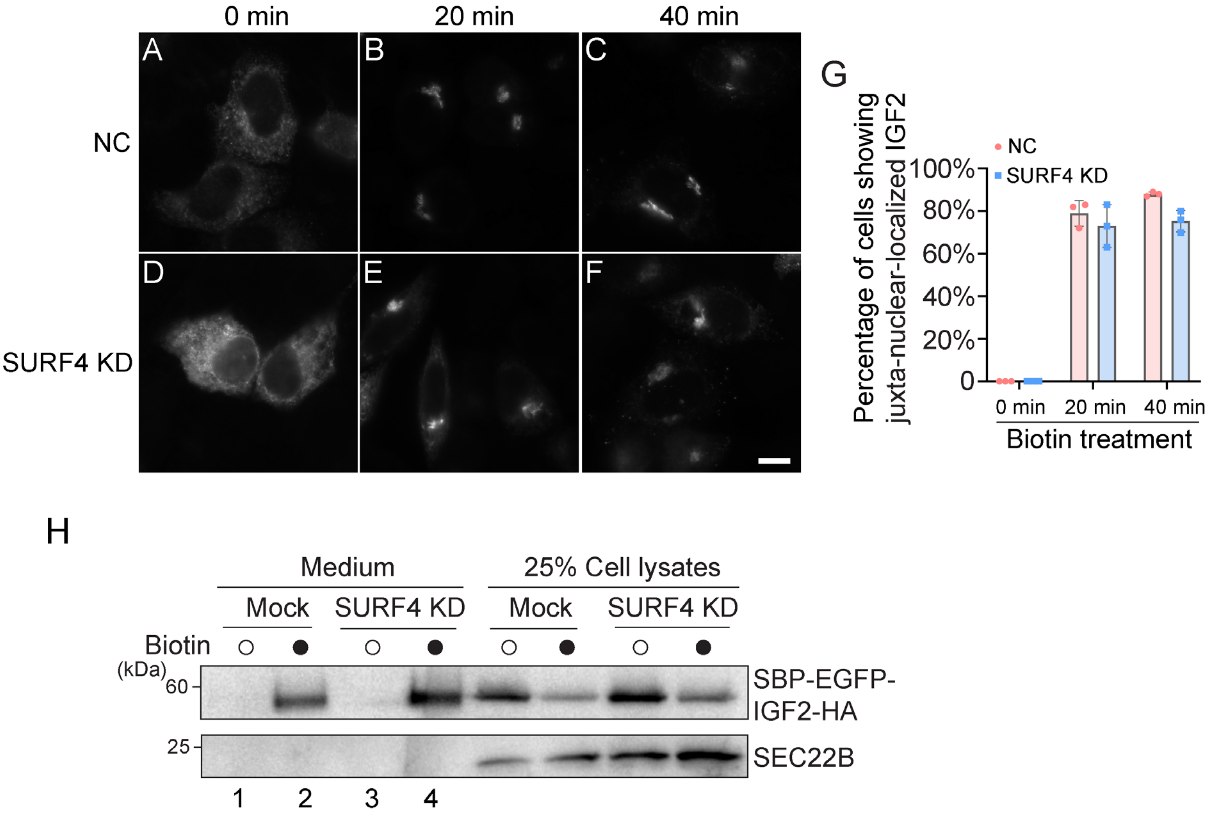

Figure S5. Knockdown of SURF4 did not cause defects in ER-to-Golgi transport and the secretion of IGF2. A-F. HeLa cells were transfected with negative control (NC) siRNA (A-C) or siRNAs against SURF4 (D-F). 48hr after transfection, cells were re-transfected with plasmids encoding Str-KDEL_ SBP-EGFP-IGF2. On day 3 after knockdown, cells were incubated with biotin for the indicated time and the localization of IGF2 was analyzed. *Scale bar,* 10 µm. *Magnification,* 63x. **G.** Quantification of the percentage of cells showing juxta-nuclear-localized SBP-EGFP-IGF2 (n = 3, mean ± S.D., over 100 cells were quantified in each experimental group). **H.** HEK293T cells were transfected with control siRNA or siRNA against SURF4. 24hr after transfection, cells were re-transfected with plasmids encoding Str-KDEL and SBP-EGFP-IGF2-HA. On day 3 after knockdown, cells were incubated with biotin for 2 hr. After biotin incubation, the level of SBP-EGFP-IGF2-HA in the medium and in cell lysates was analyzed by immunoblotting with anti-HA or anti-SEC22B antibodies.

Figure S6

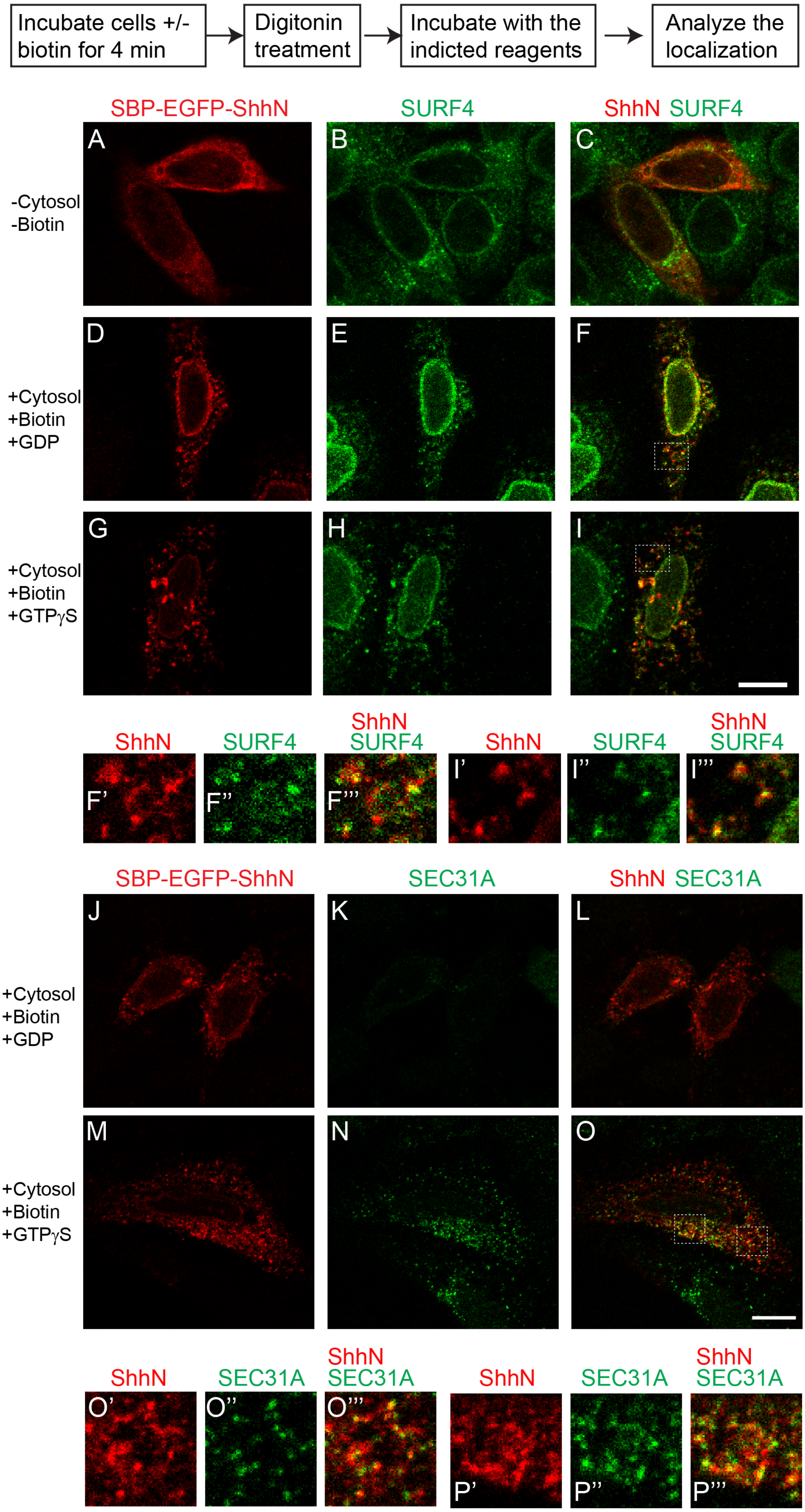

Incubate cells +/- biotin for 4 min
Digitonin treatment
Incubate with the indicted reagents
Analyze the localization
SBP-EGFP-ShhN
SURF4
ShhN SURF4
A
B
C
-Cytosol
-Biotin
D
E
F
+Cytosol
+Biotin
+GDP
G
H
I
+Cytosol
+Biotin
+GTPγS
ShhN
SURF4
ShhN SURF4
F'
F''
F'''
I'
I''
I'''
SBP-EGFP-ShhN
SEC31A
ShhN SEC31A
J
K
L
+Cytosol
+Biotin
+GDP
M
N
O
+Cytosol
+Biotin
+GTPγS
ShhN
SEC31A
ShhN SEC31A
O'
O''
O'''
P'
P''
P'''

Figure S6. Analysis of the colocalization between SBP-EGFP-ShhN and SURF4 or SEC31A using a permeabilized cell assay. A-O. HeLa cells were transfected with SBP-EGFP-ShhN$^{25\text{-}198}$ (referred to as SBP-EGFP-ShhN). Day 1 after transfection, cells were untreated (A-C) or treated with biotin for 4 min (D-O). Subsequently, cells were permeabilized by digitonin and incubated with the indicated reagents. After incubation, the localization of the indicated proteins was analyzed by immunofluorescence. Size Bar, 10 μm. Magnified views of the indicated areas in panels F and I are shown in panels F'-F''' and I'-I'''. Magnified views of the indicated areas in panel O are shown in panels O'-P'''.

Figure S7

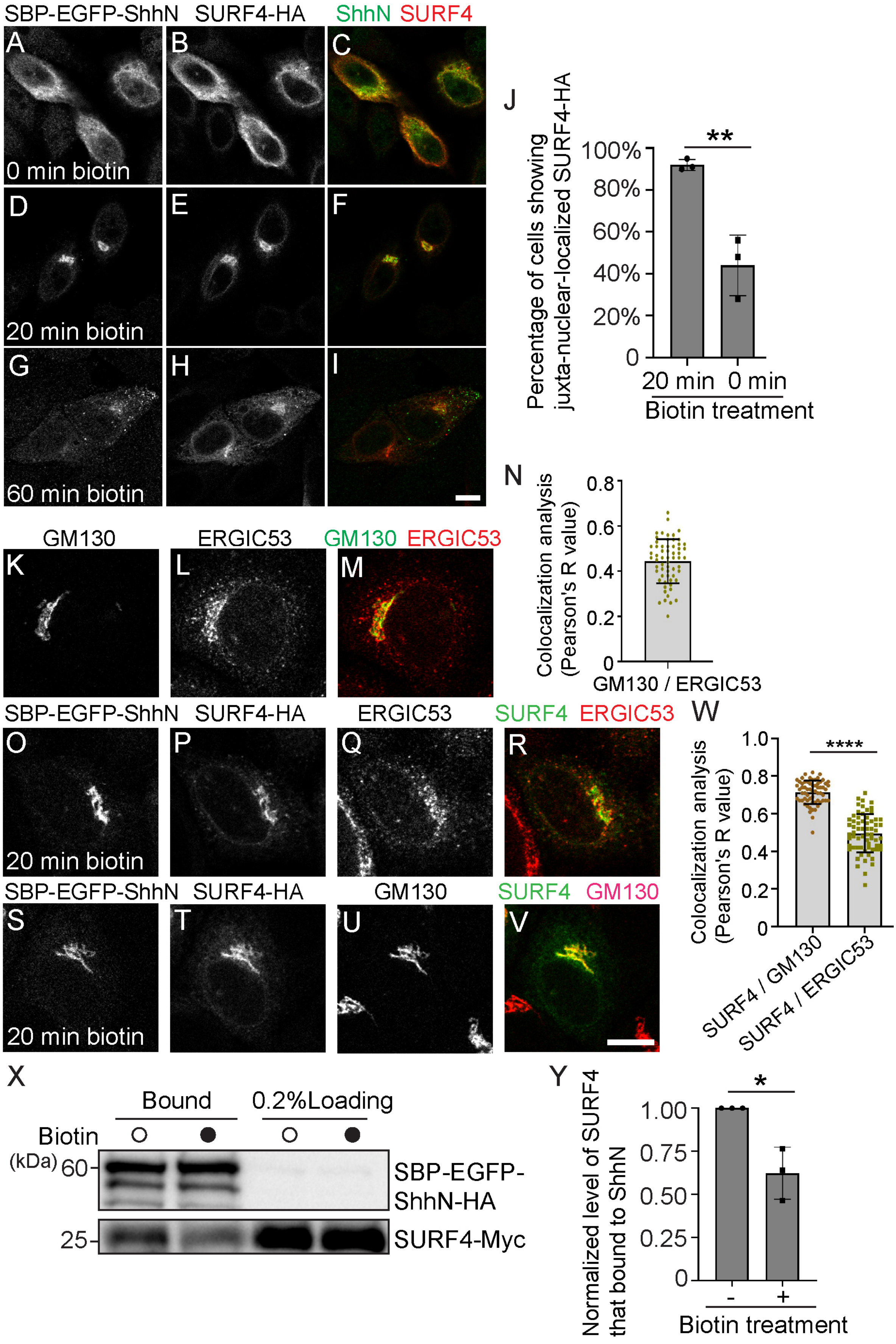
SBP-EGFP-ShhN
SURF4-HA
ShhN SURF4
A
B
C
0 min biotin
D
E
F
20 min biotin
G
H
I
60 min biotin
J
Percentage of cells showing juxta-nuclear-localized SURF4-HA
100%
80%
60%
40%
20%
0
**
20 min
0 min
Biotin treatment
GM130
ERGIC53
GM130 ERGIC53
K
L
M
N
Colocalization analysis (Pearson's R value)
0.8
0.6
0.4
0.2
0
GM130 / ERGIC53
SBP-EGFP-ShhN
SURF4-HA
ERGIC53
SURF4 ERGIC53
O
P
Q
R
20 min biotin
SBP-EGFP-ShhN
SURF4-HA
GM130
SURF4 GM130
S
T
U
V
20 min biotin
W
Colocalization analysis (Pearson's R value)
1.0
0.8
0.6
0.4
0.2
0
****
SURF4 / GM130
SURF4 / ERGIC53
X
Bound
0.2%Loading
Biotin
(kDa) 60
25
SBP-EGFP-ShhN-HA
SURF4-Myc
Y
Normalized level of SURF4 that bound to ShhN
1.00
0.75
0.50
0.25
0
*
-
+
Biotin treatment

Figure S7. SURF4 traffics together with ShhN from the ER to the Golgi.

A-I. HeLa cells were co-transfected with SBP-EGFP-ShhN$^{25\text{-}198}$ (SBP-EGFP-ShhN) and SURF4-HA. Day 1 after transfection, the localizations of the indicated proteins were analysed 0 min (A-C), 20 min (D-F) or 60 min (G-I) after biotin treatment. **J.** Quantification of the percentage of cells showing Golgi-localized SURF4-HA in cells co-expressing SURF4-HA and SBP-EGFP-ShhN 0 min or 20 min after biotin treatment (n = 3, mean ± S.D., over 100 cells were quantified in each experimental group). **, $p < 0.01$. **K-M.** The localizations of ERGIC53 and GM130 were analyzed in HeLa cells. **N.** Quantification of the colocalization between GM130 and ERGIC53 (n = 3, mean ± S.D., over 20 cells were quantified in each experimental group). **O-V.** HeLa cells co-transfected with SURF4-HA and SBP-EGFP-ShhN were treated with biotin for 20 min, and the localizations of the indicated proteins were analysed. *Scale bar,* 10 µm. *Magnification,* 63x. **W.** Quantification of the colocalization between SURF4-HA and ERGIC53 or GM130 (n = 3, mean ± S.D., over 20 cells were quantified in each experimental group). ****, $p < 0.0001$. **X.** HEK293T cells were co-transfected with plasmids encoding SURF4-Myc and SBP-EGFP-ShhN-HA. Day 1 after transfection, cells were incubated at 20°C for 2 hr in the absence or presence of biotin. Then the cells were treated in 2 mM DSP, and the cell lysates were incubated with beads conjugated with HA antibodies. The bound proteins were analyzed by immunoblotting with anti-HA or anti-Myc antibodies. **Y.** Relative levels of SURF4-Myc that bound to SBP-EGFP-ShhN-HA were quantified (n = 3, mean ± S.D.). *, $p < 0.05$.

Figure S8

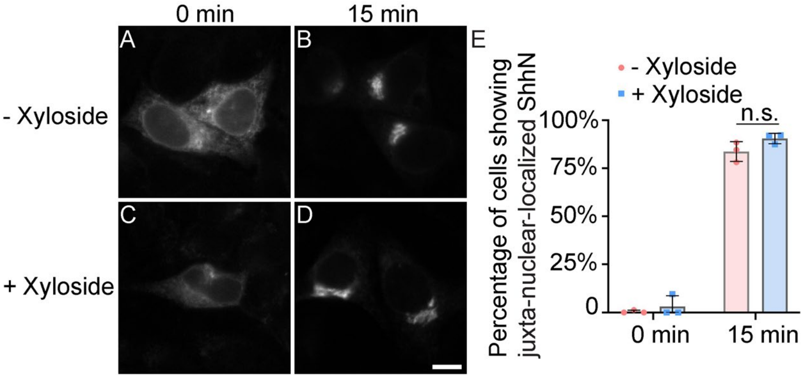

Figure S8. Xyloside treatment did not cause defects in ER-to-Golgi transport of ShhN.

A-D. HeLa cells were untreated or treated with 2.5 mM xyloside. 24hr after xyloside treatment, cells were transfected with plasmids encoding Str-KDEL and SBP-EGFP-ShhN$^{25\text{-}198}$ (SBP-EGFP-ShhN). 48 hr after xyloside treatment, cells were incubated with biotin for the indicated time. After biotin incubation, the localization of SBP-EGFP-ShhN was analyzed. *Scale bar,* 10 µm. *Magnification,* 63x. **E.** Quantification of the percentage of cells showing juxta-nuclear-localized SBP-EGFP-ShhN (n = 3, mean ± S.D., over 100 cells were quantified in each experimental group). n.s., non-significant.

Figure S9

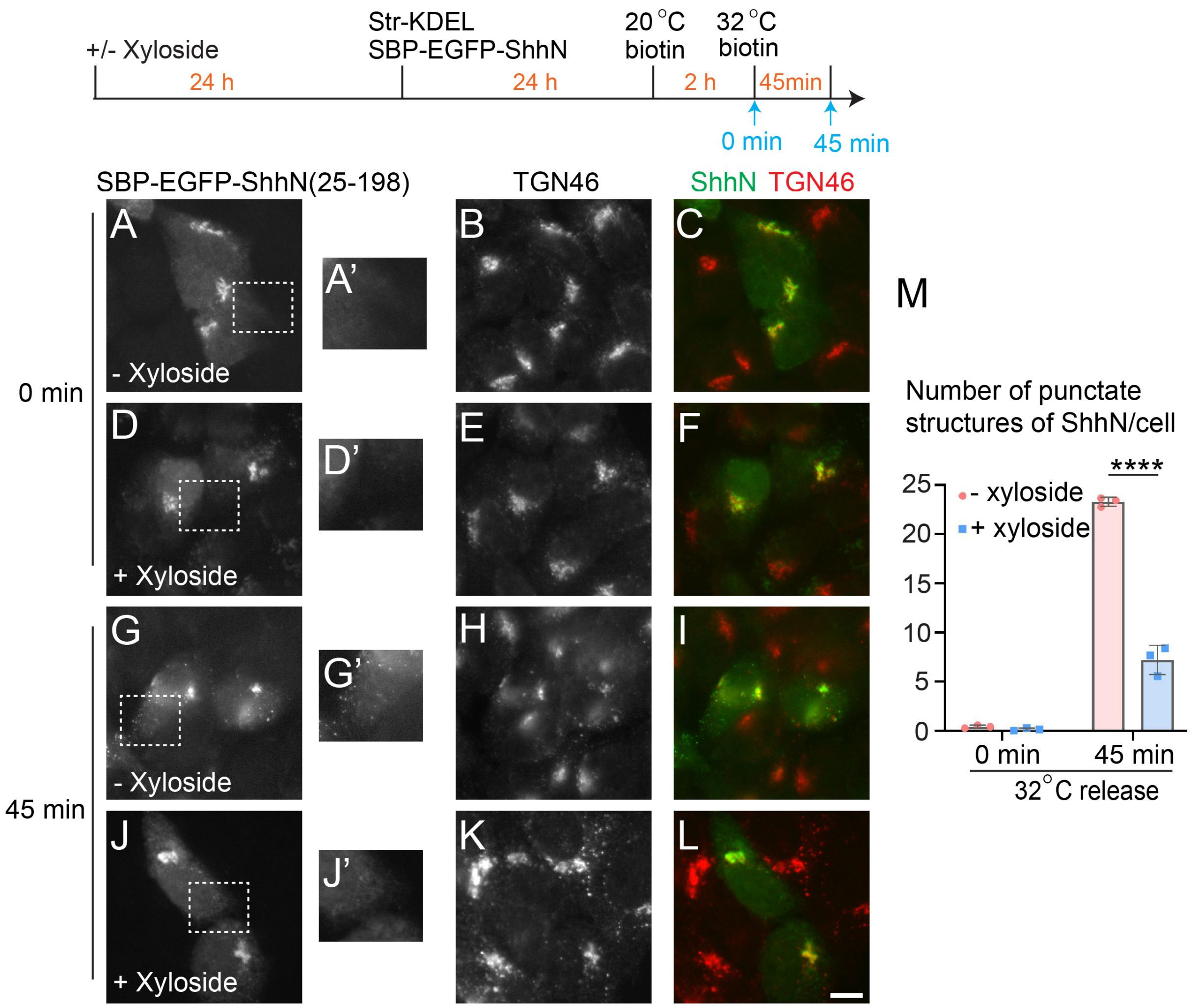

Figure S9. Inhibition of PG synthesis by xyloside causes defects in TGN export of ShhN.

A-L. HeLa cells were treated (or not) with 2.5 mM xyloside. 24hr after xyloside treatment, cells were transfected with plasmids encoding Str-KDEL and SBP-EGFP-ShhN$^{25-198}$. 48 hr after xyloside treatment, cells were treated with biotin and incubated at 20°C for 2 hr. Then the cells were incubated at 32°C for 0min (A-F) or 45 min (G-L). The localizations of SBP-EGFP-ShhN$^{25-198}$ and TGN46 were analysed. *Scale bar,* 10µm. *Magnification,* 63x. The magnified views of the indicated areas in panels A, D, G, J are shown in panels A', D', G', J'. **M.** Quantification of the number of punctate structures labelled with SBP-EGFP-ShhN$^{25-198}$ in each expressing cell (n = 3, mean ± S.D., over 25 cells were quantified in each experimental group). ****, $p < 0.0001$.

Figure S10

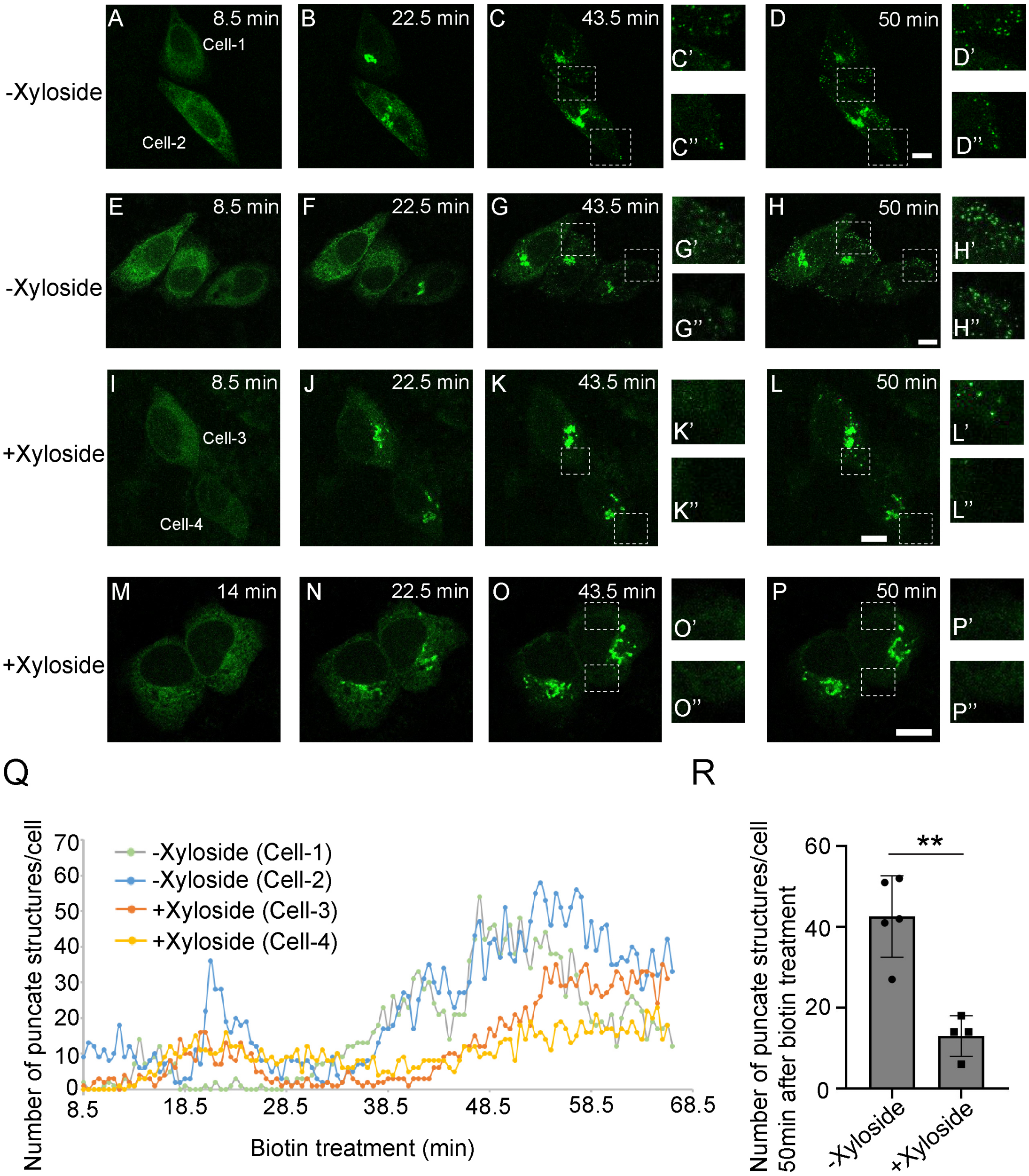

Figure S10. Live imaging analysis of the surface delivery of SBP-EGFP-ShhN.

A-P''. HeLa cells were untreated or treated with 2.5 mM xyloside. 24hr after xyloside treatment, cells were transfected with plasmids encoding Str-KDEL and SBP-EGFP-ShhN$^{25-198}$ (SBP-EGFP-ShhN). 48 hr after xyloside treatment, a time-lapse series of confocal images of SBP-EGFP-ShhN following biotin addition were acquired at an interval of 30 seconds. Representative images at selected time points are shown. The magnified views of the indicated areas in panels C-D, G-H, K-L, and O-P are shown in panels C'-D'', G'-H'', K'-L'', O'-P''. *Scale bar,* 10 µm. *Magnification,* 63x. **Q.** Quantifications of number of punctate structures of SBP-EGFP-ShhN per cell at different time points after biotin treatment. **R.** Quantifications of number of punctate structures containing SBP-EGFP-ShhN per cell at 50min after biotin treatment (mean ± S.D., each dot represents one cell). **, p<0.01.

Figure S11

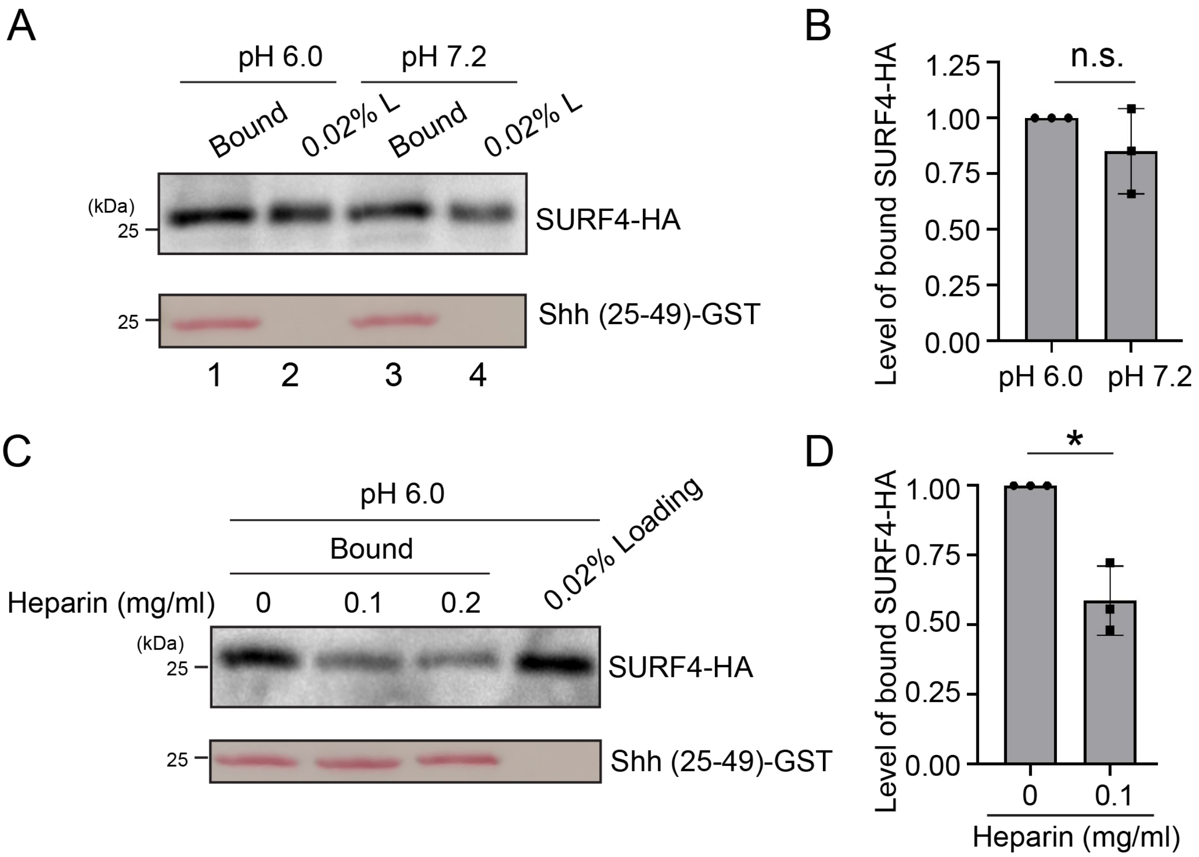

Figure S11. Lowing the pH to 6.0 did not promote the release of SURF4 from ShhN. A. Purified ShhN$^{25-49}$-GST was incubated with lysates from HEK293T cells transfected with SURF4-HA at pH 6.0 or pH 7.2. After incubation, the bound proteins were analyzed by immunoblotting with anti-HA antibodies. **B.** Relative levels of SURF4-HA that bound to ShhN$^{25-49}$-GST were quantified (n = 3, mean ± S.D.). The level of SURF4-HA that bound to ShhN$^{25-49}$-GST at pH7.2 was normalized to the corresponding bait protein, and this value was then normalized to the level of SURF4-HA that bound to ShhN$^{25-49}$-GST at pH6.0 in each experimental group. n.s., not significant. **C.** Purified ShhN$^{25-49}$-GST was incubated with lysates from HEK293T cells transfected with SURF4-HA in the presence of the indicated concentrations of heparin at pH6.0. After incubation, the bound proteins were analyzed by immunoblotting with anti-HA antibodies. **D.** Relative levels of SURF4-HA that bound to ShhN$^{25-49}$-GST were quantified (n = 3, mean ± S.D.). The quantification is normalized to the level of SURF4-HA that bound to ShhN$^{25-49}$-GST in the absence of Heparin in each experimental group. *, $p < 0.05$.

Figure S12

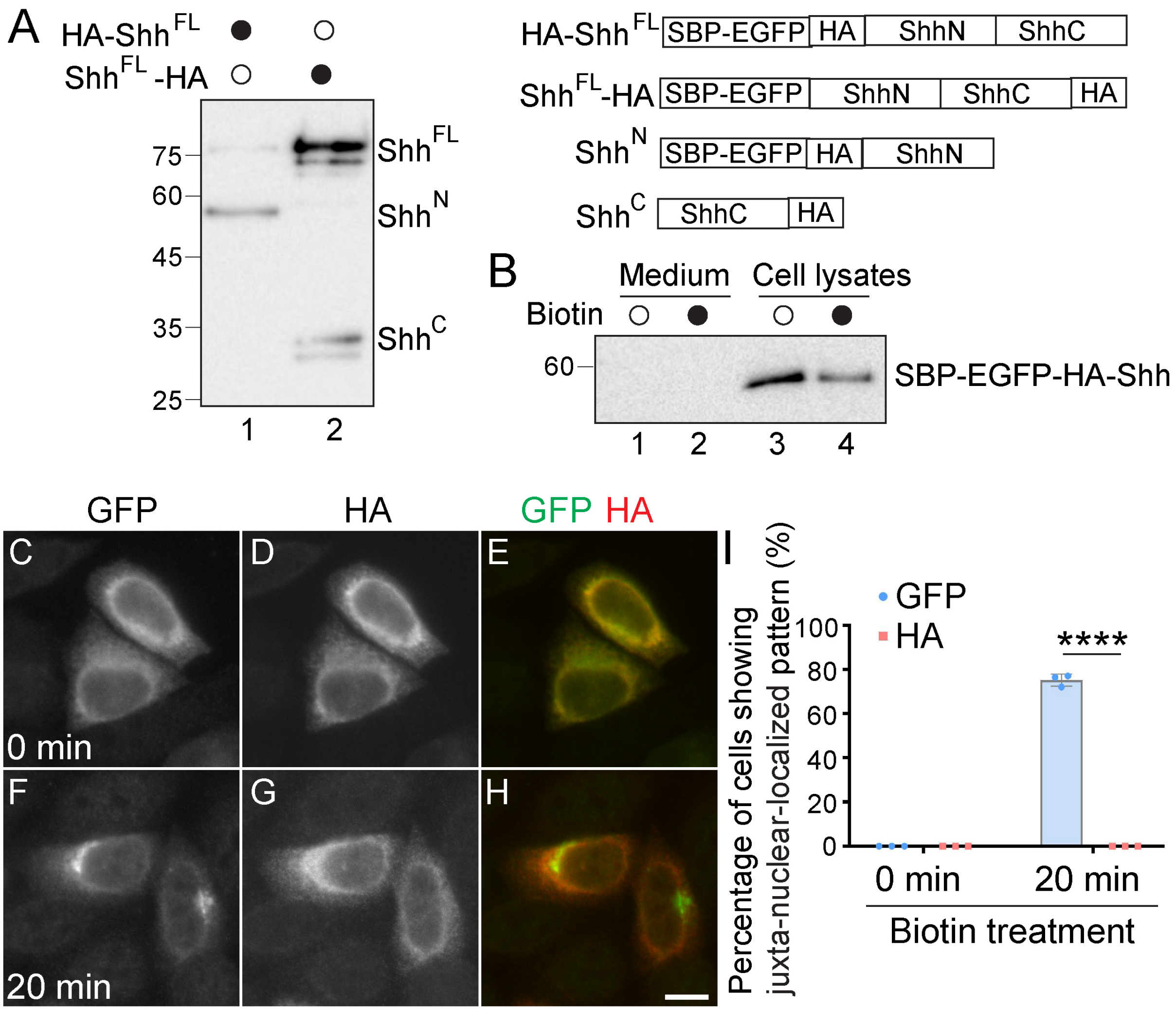

Figure S12. SBP-EGFP-Shh^{FL} is processed into N-terminal and C-terminal fragments.

A. HA-tagged Shh^{FL} in cell lysates from HEK293T cells expressing SBP-EGFP-HA-Shh^{FL} (HA-Shh^{FL}) or SBP-EGFP-Shh^{FL}-HA (Shh^{FL}-HA) was analysed by immunoblotting using anti-HA antibodies. **B.** HEK293T cells transfected with plasmids encoding Str-KDEL and SBP-EGFP-HA-Shh^{FL} were incubated in the presence or absence of biotin for 2 hr. After incubation, the level of Shh in the medium and in cell lysates was then analyzed by immunoblotting using anti-HA antibodies. **C-H.** HeLa cells were transfected with plasmids encoding Str-KDEL and SBP-EGFP-Shh^{FL}-HA. Day 1 after transfection, the localizations of the indicated proteins were analysed 0 min (C-E) or 20 min (F-H) after biotin treatment. *Scale bar,* 10 µm. *Magnification,* 63x. **I.** Quantification of the percentage of cells showing juxta-nuclear-localized SBP-EGFP-ShhN and ShhC-HA 0 min or 20 min after biotin treatment (n = 3, mean ± S.D., over 100 cells were quantified in each experimental group). ****, $p < 0.0001$.

Figure S13

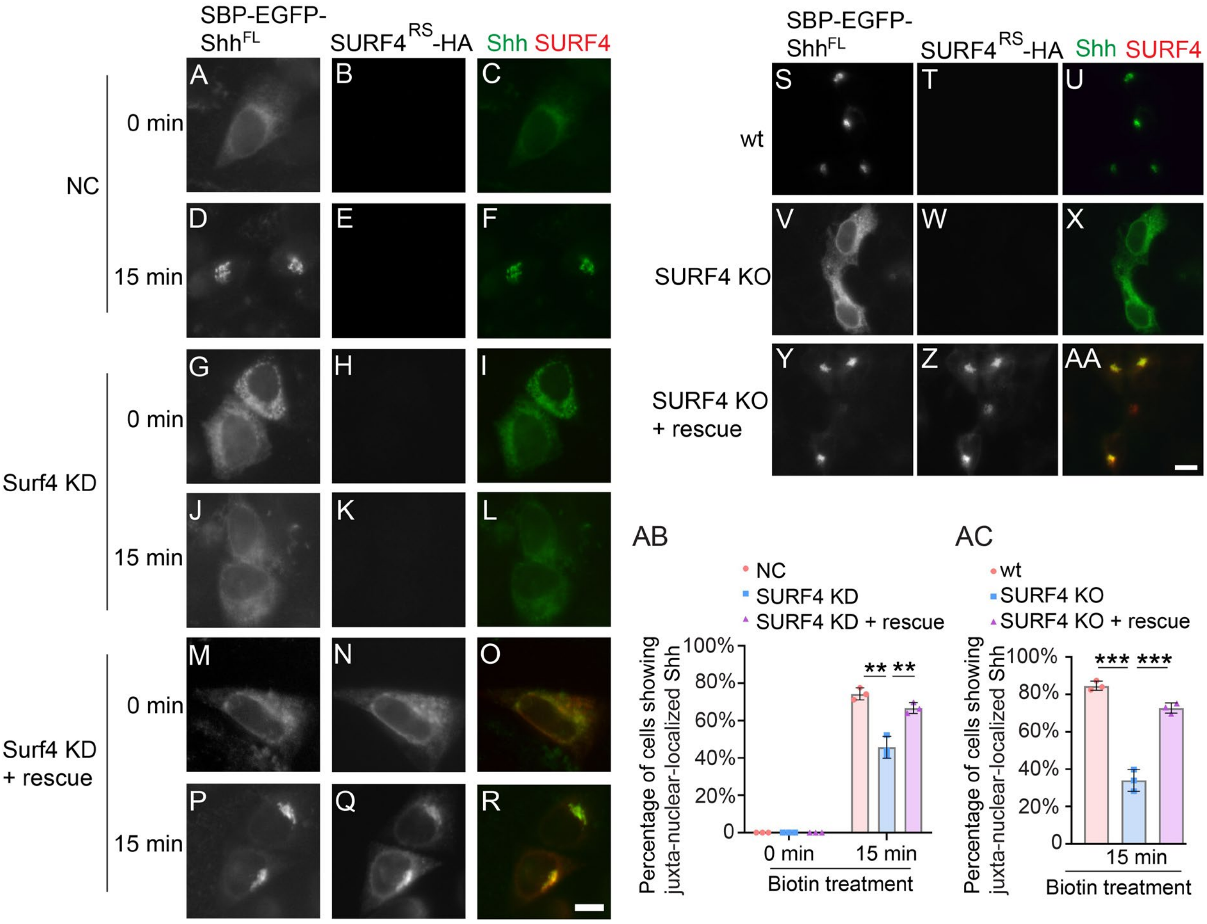

Figure S13. SURF4 regulates ER-to-Golgi transport of SBP-EGFP-Shh^{FL}.

A-R. HeLa cells were transfected with negative control (NC) siRNA (A-F) or siRNAs against SURF4 (G-R). 48hr after transfection, cells were re-transfected with plasmids encoding Str-KDEL_ SBP-EGFP-Shh^{FL} (A-L), or siRNA-resistant SURF4^{RS}-HA and Str-KDEL_ SBP-EGFP-Shh^{FL} (M-R). On day 3 after knockdown, cells were incubated with biotin for the indicated time and the localization of Shh was analyzed. **S-AA.** Wild type (wt) or SURF4 KO HEKTrex cells were transfected with plasmids encoding Str-KDEL_ SBP-EGFP-Shh^{FL} (S-X), or re-transfected with plasmids encoding the siRNA-resistant SURF4^{RS}-HA and Str-KDEL_ SBP-EGFP-Shh^{FL} (Y-AA). On day 3 after knockdown, cells were incubated with biotin for 15 min and the localizations of the indicated proteins were analyzed. *Scale bar,* 10 µm. *Magnification,* 63x. **AB-AC.** Quantification of the percentage of cells showing juxta-nuclear-localized SBP-EGFP-Shh^{FL} (n = 3, mean ± S.D., over 100 cells were quantified in each experimental group). **, $p < 0.01$; ***, $p < 0.001$.

Figure S14

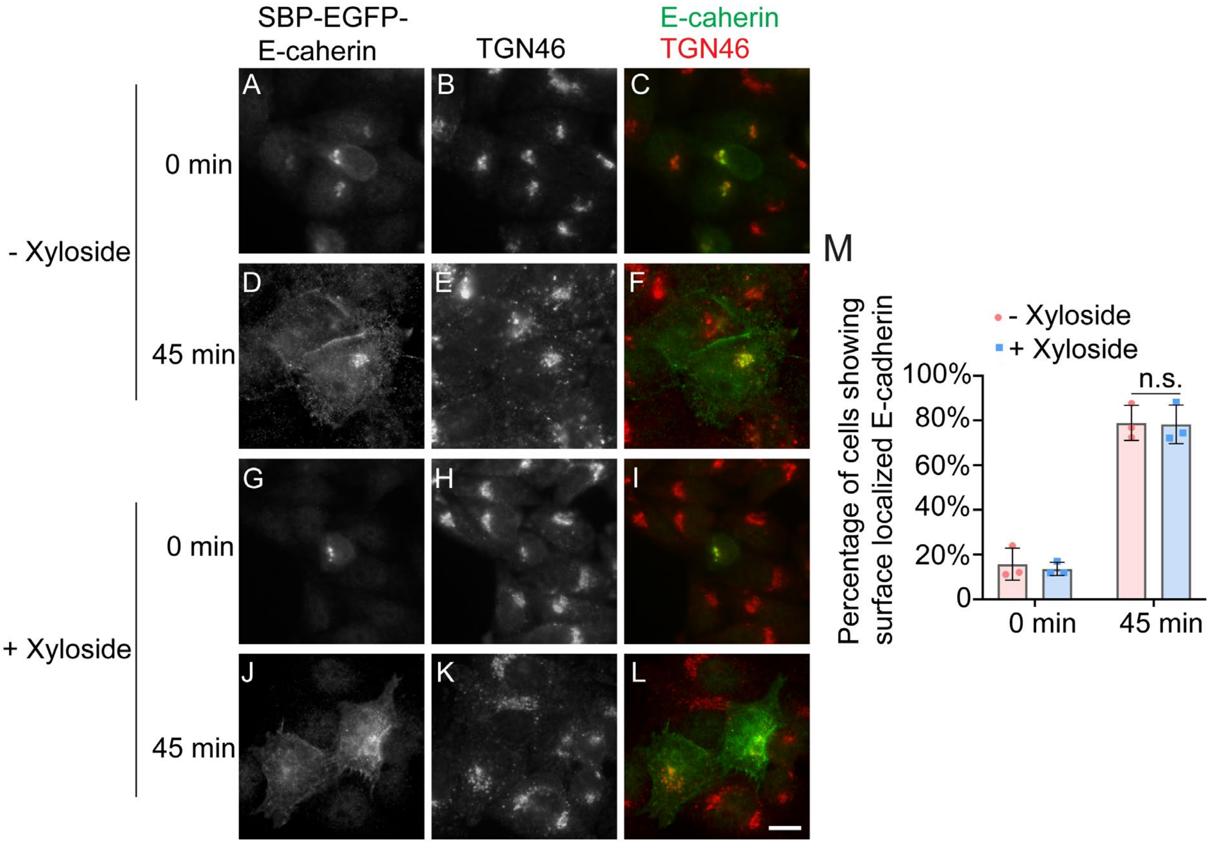

Figure S14. Blocking synthesis of proteoglycan does not inhibit the surface delivery of SBP-EGFP-E-cadherin. A-L. HeLa cells were untreated (A-F) or treated with 2.5 mM xyloside (G-L). 24hr after xyloside treatment, cells were transfected with plasmids encoding SBP-EGFP-E-cadherin. On day 3 after xyloside treatment, cells were treated with biotin and incubated in the 20°C for 2 hr. Then the cells were incubated in 32°C for 0min (A-C, G-I) or 45 min (D-F, J-L). The localizations of the indicated constructs were analysed. *Size bar,* 10μm. **M.** Quantifications of the percentage of cells showing surface-localized SBP-EGFP-E-cadherin (n = 3, mean ± S.D., over 100 cells were quantified in each experimental group). n.s. not significant.

Movie 1

A time-lapse video of SBP-EGFP-ShhN in HeLa cells untreated with xyloside. This video was generated from a time-lapse series of confocal images of SBP-EGFP-ShhN following biotin addition at an interval of 30 seconds. Representative frames are shown in Figure S10.

Movie 2

A time-lapse video of SBP-EGFP-ShhN in HeLa cells untreated with xyloside. This video was generated from a time-lapse series of confocal images of SBP-EGFP-ShhN following biotin addition at an interval of 30 seconds. Representative frames are shown in Figure S10.

Movie 3

A time-lapse video of SBP-EGFP-ShhN in HeLa cells treated with 2.5 mM xyloside. This video was generated from a time-lapse series of confocal images of SBP-EGFP-ShhN following biotin addition at an interval of 30 seconds. Representative frames are shown in Figure S10.

Movie 4

A time-lapse video of SBP-EGFP-ShhN in HeLa cells treated with 2.5 mM xyloside. This video was generated from a time-lapse series of confocal images of SBP-EGFP-ShhN following biotin addition at an interval of 30 seconds. Representative frames are shown in Figure S10.
